# Aging and memory are altered by genetically manipulating lactate dehydrogenase in the neurons or glia of flies

**DOI:** 10.18632/aging.204565

**Published:** 2023-02-25

**Authors:** Ariel K. Frame, J. Wesley Robinson, Nader H. Mahmoudzadeh, Jason M. Tennessen, Anne F. Simon, Robert C. Cumming

**Affiliations:** 1 Department of Biology, Western University, London, Canada; 2 Department of Biology, Indiana University, Bloomington, IN 47405, USA

**Keywords:** astrocyte-neuron lactate shuttle (ANLS), lactate, lactate dehydrogenase, dLdh, *Drosophila melanogaster*, glia, long-term memory, courtship conditioning

## Abstract

The astrocyte-neuron lactate shuttle hypothesis posits that glial-generated lactate is transported to neurons to fuel metabolic processes required for long-term memory. Although studies in vertebrates have revealed that lactate shuttling is important for cognitive function, it is uncertain if this form of metabolic coupling is conserved in invertebrates or is influenced by age. Lactate dehydrogenase (Ldh) is a rate limiting enzyme that interconverts lactate and pyruvate. Here we genetically manipulated expression of *Drosophila melanogaster* lactate dehydrogenase (dLdh) in neurons or glia to assess the impact of altered lactate metabolism on invertebrate aging and long-term courtship memory at different ages. We also assessed survival, negative geotaxis, brain neutral lipids (the core component of lipid droplets) and brain metabolites. Both upregulation and downregulation of dLdh in neurons resulted in decreased survival and memory impairment with age. Glial downregulation of dLdh expression caused age-related memory impairment without altering survival, while upregulated glial dLdh expression lowered survival without disrupting memory. Both neuronal and glial dLdh upregulation increased neutral lipid accumulation. We provide evidence that altered lactate metabolism with age affects the tricarboxylic acid (TCA) cycle, 2-hydroxyglutarate (2HG), and neutral lipid accumulation. Collectively, our findings indicate that the direct alteration of lactate metabolism in either glia or neurons affects memory and survival but only in an age-dependent manner.

## INTRODUCTION

The brain is an energetically demanding organ relative to other tissues, a phenomenon found in multiple species from humans to flies [1–4]. Various metabolites can be utilized to meet the energy requirements of neural cells, although the preferred metabolite may differ by cell-type, brain region, age, and behavioural context. Over the last two decades there has been growing recognition that lactate, the end product of glycolysis, serves many functions, including acting as a source of energy, a signaling molecule, and even as an epigenetic regulator. The high metabolic demands of the brain and the numerous roles of lactate in CNS function have been extensively discussed elsewhere [5–10].

Multiple studies on the brains of vertebrates have lent support to the hypothesis that astrocytes supply neurons with lactate for their energetic needs [11–22]. This type of metabolic coupling is commonly referred to as the astrocyte-neuron lactate shuttle (ANLS) [23, 24]. Despite the accumulation of a large body of evidence supporting the existence of an ANLS, there is still controversy surrounding the physiological role and importance of the ANLS [25–31]. How the ANLS contributes to behaviour has yet to be fully elucidated. Most studies investigating the role that the ANLS plays in behaviour have focused on mammals. However, astrocyte function is also conserved in invertebrates [32–35]. In fact, evidence for a glia-neuron metabolic coupling in invertebrates actually preceded the discovery of the ANLS [36–38]. Recent studies suggest that invertebrate animals such as *Drosophila melanogaster* rely on glia to produce and release lactate, through monocarboxylate transporters, to support neuronal survival, synaptic function, axonal regeneration, and synthesis of fatty acids in a manner analogous to the ANLS in mammals [39–43]. These studies revealed that aberrant lactate metabolism in *D. melanogaster* can contribute to neurodegeneration, in part, due to neuronal mitochondrial dysfunction [41], potentiation of amyloid beta toxicity [44], and aging [45]. Age-related neurodegeneration is commonly associated with cognitive deficits, yet none of the previous studies investigated whether central nervous system (CNS) lactate metabolism impacts cognition or age-related cognitive decline in *D. melanogaster*. Therefore, *D. melanogaster* serves as a good model for understanding the role of glia-neuron lactate shuttling in central nervous system (CNS) function and cognitive behaviour.

The ANLS has been implicated in multiple cognitive processes in mammals [46–54]. One common method of demonstrating ANLS involvement in cognition involves direct infusion of a glycogenolysis inhibitor in the brain to prevent astrocytes from producing lactate from glycogen stores. Glycogenolysis inhibition causes a disruption of taste aversion memory consolidation in young chickens [46], long-term (but not short-term) inhibitory avoidance memory in rats [47], spontaneous alternation in rats [48], cocaine-induced conditioned place preference memory in rats [49, 50], and novel object recognition memory in mice [52]. Another method of determining whether the ANLS contributes to cognition involves pharmacological or genetic inhibition of monocarboxylate transporters in neurons (MCT2) or astrocytes (MCT1, MCT4) to prevent lactate shuttling. Interference with lactate transport between astrocytes and neurons leads to impaired object recognition memory in rats [54] and long-term inhibitory avoidance memory in both mice [51] and rats [47]. In contrast, relatively few studies have examined the effect of glia-neuron metabolic coupling on cognition in *D. melanogaster* [55–57]. Two recent findings suggest that *D. melanogaster,* in certain contexts, require glia to provide glucose or ketone bodies to fuel neurons, irrespective of lactate, and facilitate memory formation [55, 56]. Moreover, findings by our laboratory and others have provided evidence that glia-neuron lactate shuttling in *D. melanogaster* impacts aging and neurodegeneration [41, 44, 45]. In addition, glia have been shown to contribute to age-related memory impairment [58, 59]. Nonetheless, the contribution of glia-neuron lactate shuttling to age-related changes in *D. melanogaster* memory has not been investigated.

Lactate dehydrogenase (LDH) is a tetrameric enzyme which catalyzes the interconversion of lactate and pyruvate coupled with the exchange of the oxidized and reduced forms of the coenzyme nicotinamide adenine dinucleotide (NAD) [Pyruvate + NADH + H^+^ ↔ Lactate + NAD^+^]. This reaction serves as a central node for balancing potential outcomes of carbohydrate metabolism. The breakdown of circulating sugars such as glucose, or trehalose in the case of *D. melanogaster*, is biased towards the production of pyruvate, which can be further processed by different metabolic enzymes [60]. Pyruvate can be oxidized by the pyruvate dehydrogenase complex to acetyl coenzyme A (Acetyl-CoA), followed by subsequent oxidization into carbon dioxide (CO_2_) through the tricarboxylic acid (TCA) cycle in mitochondria. Alternatively, pyruvate can be reduced to lactate by LDH and serve as a reservoir for oxidative metabolism later following the conversion back into pyruvate by LDH [61]. Therefore, glia rely on LDH to generate lactate, which is subsequently used as a fuel source by neurons following LDH-mediated conversion from lactate to pyruvate to fuel oxidative phosphorylation. Vertebrates have two isoforms of LDH expressed in the brain, LDHA and LDHB, which are believed to be differentially expressed in glia and neurons [62, 63]. Five different LDH isozymes can be formed depending on which vertebrate LDH isoforms make up the subunit composition of the LDH tetramer (LDH-1 = B4, LDH-2 = B1A3, LDH-3 = B2A2, LDH-4 = B1A3, LDH-5 = A4). The enzyme kinetics of LDH isozymes differ such that those containing more LDHA favor conversion of pyruvate to lactate and those containing more LDHB favor the conversion of lactate to pyruvate [64–73]. In contrast, *D. melanogaster* LDH is a tetramer made up of four identical subunits encoded by a single LDH homolog gene *ImpL3/dLdh* (FBgn0001258) [74, 75] that is expressed in glia and neurons [76, 77]. Interestingly, in neuronal nuclei of the mushroom body brain region, the main olfactory memory centre within *D. melanogaster*, *dLdh* mRNA levels are nearly 8-fold higher compared to the rest of the brain [77]. Moreover, aging in *D. melanogaster* causes an increase in *dLdh* mRNA [45, 78–80] and dLdh protein levels in the brain [45]. In addition, adult specific *dLdh* overexpression, either ubiquitously or in a tissue specific manner in muscle, neurons, and glia, has recently been shown to decrease lifespan [45, 81]. Nonetheless, the impact of altered *dLdh* expression in specific CNS cell types on memory, in an aging context, has not yet been examined in flies.

In *D. melanogaster*, age-related decline in cognitive function has been well documented [82, 83]. One of the most utilized classical conditioning assays for assessing *D. melanogaster* learning and memory is the aversive olfactory associative learning task that requires exposing flies to two separate odors; one paired with electric shocks and the other without, then measuring preference for the non-paired odor between the two [84]. Olfactory associative memory is particularly sensitive to aging [85, 86] and requires various types of metabolism depending on the training and retrieval conditions [55–57, 87, 88]. Another well characterized assay for assessing *D. melanogaster* learning and memory is the courtship conditioning paradigm. Courtship conditioning occurs when rejection by females of male courtship attempts promotes a reduction of future male courtship attempts [89]. Courtship memory is similar to aversive olfactory associative memory in that both behaviours require olfaction, depend on the mushroom body region of the brain, and can be retained for days upon appropriate training conditions [90–92].

Here we studied the effect of genetic alteration of *dLdh* expression within adult neurons or glia on long-term courtship memory and aging. We first show that long-term courtship memory is unaffected with age in our control flies (Canton-S); a finding in agreement with previous studies of short-term courtship memory [93, 94] and in contrast to previous studies assessing olfactory based long-term memory [85, 86]. However, we found that genetic changes in lactate metabolism result in alterations in long-term courtship memory in an age- and cell-type-dependent manner. We provide evidence that both increasing and decreasing *dLdh* expression within neurons negatively affects survival and maintenance of long-term courtship memory with age. Paradoxically, excess glial *dLdh* expression reduces lifespan without a change in memory, whereas decreased glial *dLdh* expression causes an age-dependent deficit in long-term courtship memory in the absence of any effect on lifespan. Furthermore, we show that changes in metabolite levels and neutral lipid accumulation may underlie age-related impacts of altered lactate metabolism, suggesting that altered glia-neuron lactate and lipid shuttling contribute to cognitive aging and decreased brain health in *D. melanogaster*.

## RESULTS

### Lactate dehydrogenase expression and long-term courtship memory is maintained with age in Canton-S flies

**Figure 1:**
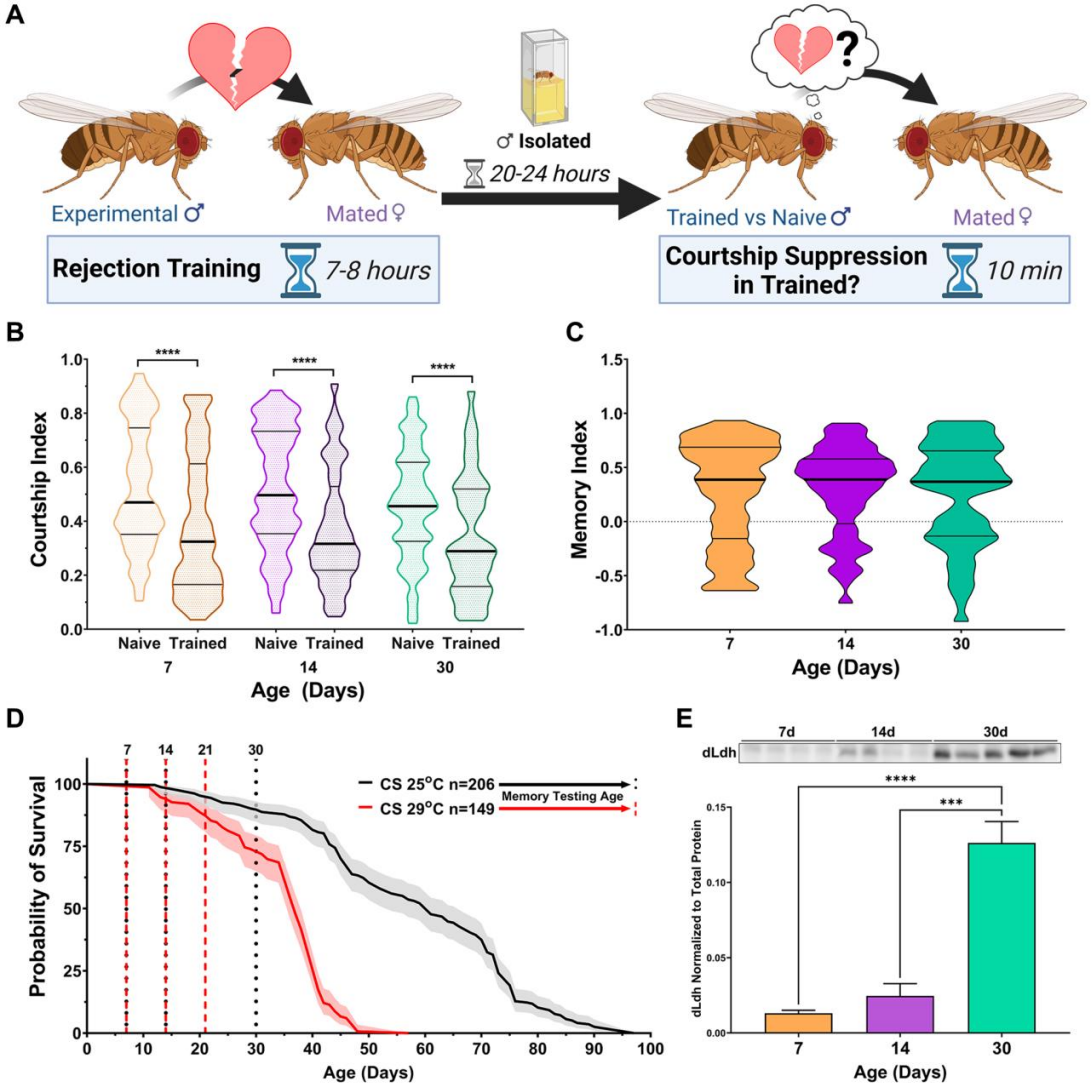
***dLdh* protein levels increase in the brain with age while long-term courtship memory is retained in control male flies.** (**A**) Courtship conditioning paradigm used for testing long-term courtship memory throughout this study. (**B**) Courtship indexes for Canton-S male flies aged 7, 14, or 30 days at 25°C decreased in courtship conditioning rejection trained vs. naïve conditions at all ages. *N* = 100–117. Naïve and trained flies were compared for each age group using one-sided Mann-Whitney *U* tests, ^****^p < 0.0001. Data presented as violin plot of frequency distribution. (**C**) Long-term courtship memory indexes for trained flies represented in B do not differ between age groups. Age groups were compared using a Kruskal-Wallis test with Dunn’s multiple comparisons tests. (**D**) Survival curve of Canton-S male flies singly housed is decreased at 29°C compared with 25°C and ages which were selected for memory testing in control (25°C black) and transgenic (29°C red) flies are highlighted with vertical dotted lines. Curve comparison was made using a Log-rank (Mantel-Cox) test, ^****^p < 0.0001. Data presented as violin plot of frequency distribution. (**E**) Western blot analysis of head extracts from male Canton-S flies aged 7, 14 or 30 days at 29°C showing dLdh protein levels increase with age (*F*(2, 10) = 36.64, *p* < 0.0001) *n* = 4–5. Each sample consists of protein extracts from 20 heads. Comparisons across age were made using one-way ANOVA with Šídák’s multiple comparisons tests between all age groups. ^***^p < 0.001, ^****^p < 0.0001.

In this study, courtship conditioning was employed to assess long-term memory (Figure 1A). To date, there are no reports examining the effects of age on long-term courtship memory in a reference or control line such as Canton-S (CS). We observed that 24-hour long-term courtship memory was retained in CS up to 30 days of age under standard growth temperature conditions (25°C) (Figure 1B and 1C). These results indicate that long-term courtship memory is not susceptible to age-related impairment, at least up to 30 days of age at 25°C, a time point when 89.7% of the population is still alive.

We restricted genetic manipulation of *dLdh* expression to adulthood by employing a system which utilizes temperature sensitive Gal80 to inhibit Gal4 activity at 18°C, while permitting activity at 29°C [95]. We set up a reference longevity curve, in which CS flies were reared and assayed at 29^o^C. At this temperature, flies exhibited approximately 90% survival at 21 days of age, which is similar to CS flies reared at 25°C at 30 days of age (Figure 1D). Thus, to limit survivorship bias we selected 21 days of age raised at 29°C for analysis of aged flies. We also examined endogenous levels of dLdh at this timepoint by western blot analysis. CS flies reared at 29°C exhibited an increase in whole head dLdh protein levels with age (Figure 1E, Supplementary Figure 1), in accordance with our previous evaluation of aged cantonized *w^1118^* flies maintained at 25°C [45] and with others who have measured *dLdh* only at the transcript level [78–80, 96].

### Both increasing and decreasing neuronal lactate dehydrogenase expression is detrimental to survival and long-term courtship memory in aged flies

**Figure 2:**
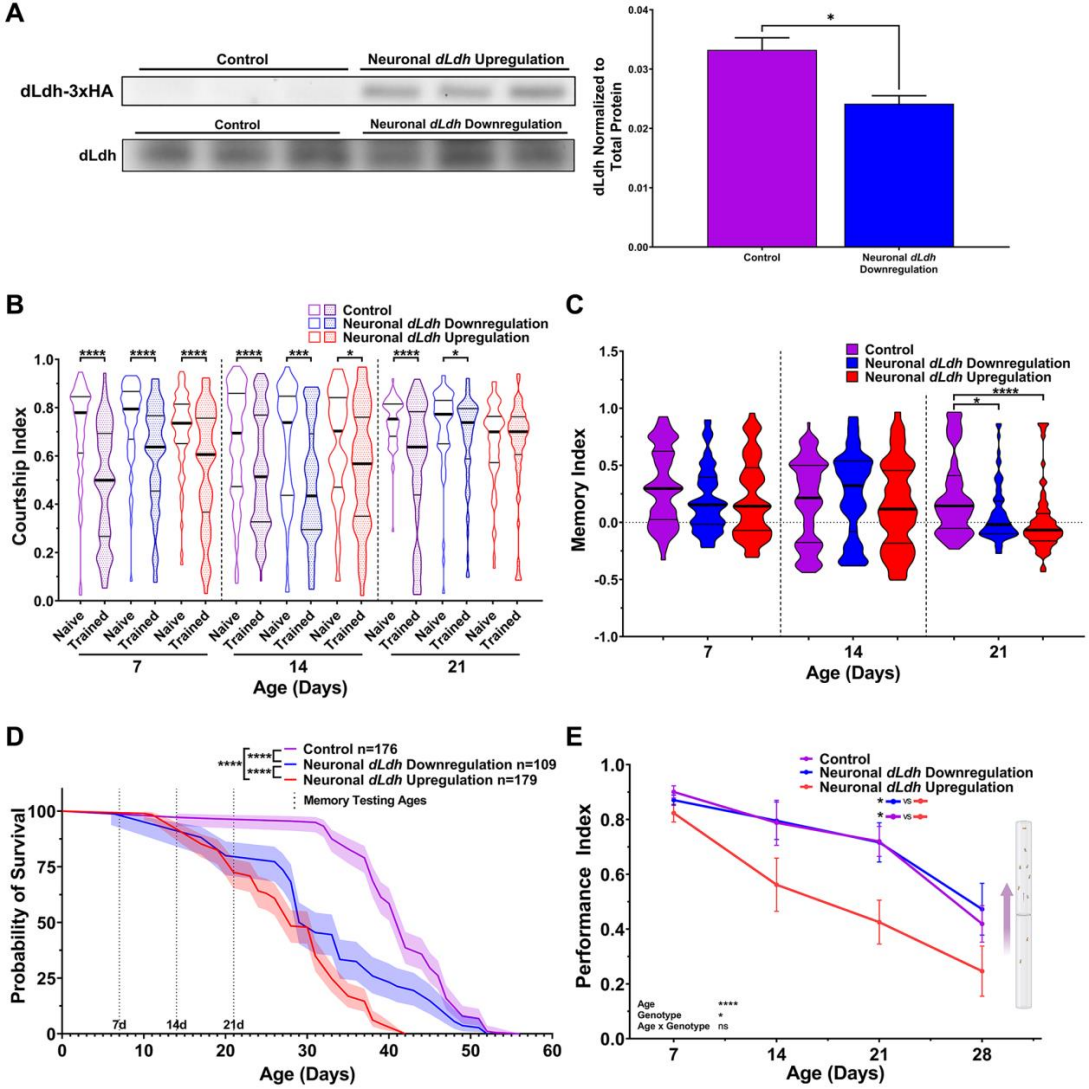
**Male flies with either elevated or downregulated neuronal *dLdh* expression exhibit decreased long-term courtship memory and survival with age**. (**A**) Western blot analysis of head extracts from neuronal transgenic male flies aged 21 days at 29°C showing elevated ectopic HA tagged dLdh expression (upper panel) and decreased endogenous dLdh levels (lower panel) in flies using a Elavts-Gal4 driver to drive UAS-dLDH (with 3xHA tag) and UAS-dLdh-RNAi expression respectively (*n* = 3), expression was normalized to total protein content (based on Ponceau-S staining). Each sample consisted of protein extracts from 20 heads. Comparison of endogenous dLdh level were made using an unpaired *t* test, ^*^p < 0.05. (**B**) Courtship indexes for neuronal transgenic male flies aged 7, 14, or 21 days at 29°C post-eclosion showed a decrease in courtship conditioning rejection trained vs. naïve for all genotypes at all ages, except for 21 day upregulation. *N* = 59–125. Naïve and trained flies were compared within each genotype at each age using one-sided Mann-Whitney *U* tests, ^****^p < 0.0001, ^***^p < 0.001, ^*^p < 0.05. Data presented as violin plot of frequency distribution. (**C**) Long-term courtship memory indexes for neuronal transgenic trained flies represented in B. Memory in neuronal transgenic flies only differed from control at 21 days of age (*H* = 21.33, *p* < 0.0001), with both flies with *dLdh* upregulation and downregulation showing reduced memory at 21 day of age compared to control. Genotypes at each age were compared using Kruskal-Wallis tests with Dunn’s multiple comparisons to control, ^****^p < 0.0001, ^*^p < 0.05. Data presented as violin plot of frequency distribution. (**D**) In male flies, both neuronal upregulation and downregulation of *dLdh* resulted in reduced survival compared to control. Shaded area represents the 95% confidence interval. Curve comparisons were made using a Log-rank (Mantel-Cox) test, ^****^p < 0.0001. Ages chosen for memory testing are highlighted with vertical dotted lines. (**E**) Upregulation of neuronal *dLdh* in male flies decreased climbing ability during negative geotaxis compared to control and *dLdh* downregulation (genotype effect *F*(2, 33) = 4.355, *p* = 0.0210). Comparisons across age and genotype were made using a mixed-effects model with Geisser-Greenhouse correction and Šídák’s multiple comparisons within each age group, ^*^p < 0.05, ^****^p < 0.0001. Effects of age, genotype, and age by genotype interaction are denoted on the bottom left.

In order to assess the effects of altered *dLdh* expression on memory in an adult context, we used flies containing a temperature sensitive Gal80 driven by a ubiquitously expressing Tubulin driver and Gal4 driven by a pan-neuronal expressing Elav driver (thereafter Elavts-Gal4). These flies were subsequently crossed with flies containing an upstream activation sequence (UAS) driving either the dLdh full-length open reading frame with a triple hemagglutinin (HA) tag on the C-terminus (UAS-dLdh) or an RNA interference construct targeting *dLdh* (UAS-dLdh-RNAi), to permit either increased or decreased expression of *dLdh* in adult neurons respectively. Firstly, we verified by western blot that dLdh protein levels decreased in the heads of neuronal *dLdh* downregulated flies and that there was induction of HA-tagged dLdh production in the heads of neuronal *dLdh* upregulated flies at 21 days of age (Figure 2A, Supplementary Figures 2 and 3). Using the same courtship conditioning paradigm utilized in CS flies, we tested long-term courtship memory in dLdh transgenic flies. At 7 and 14 days of age, neither neuronal *dLdh* upregulation nor downregulation elicited a change in long-term courtship memory (Figure 2B and 2C). However, at 21 days of age, flies with either a neuronal upregulation or downregulation of *dLdh* expression showed a decrease in long-term courtship memory compared to control flies (Figure 2B and 2C). In addition to memory, we tested whether aging affected health in neuronal *dLdh* transgenic flies by measuring survival and climbing ability. Flies with neuronal *dLdh* downregulation exhibited a clear decrease in survival compared to control flies, with an earlier onset of death, a pronounced increase in the rate of death, but an unchanged maximum lifespan (Figure 2D). Flies with neuronal *dLdh* upregulation also exhibited reduced survival compared to control flies, with a similar decrease in time of death onset and rate of death, but also with a decrease in maximum lifespan (Figure 2D). Unhealthy flies tend to exhibit a general inability to maintain locomotor behaviours as assessed using a negative geotaxis assay. We found that climbing ability was decreased in flies with neuronal *dLdh* upregulation compared to control flies at 21 days of age (Figure 2E). These findings indicate that the rate of age-related decline in climbing ability of flies with neuronal *dLdh* upregulation was more pronounced than control flies. However, flies with neuronal *dLdh* downregulation did not exhibit a change in climbing ability compared to control flies at any age (Figure 2E).

### Altered glial lactate dehydrogenase expression promotes differential effects on survival and long-term courtship memory

**Figure 3:**
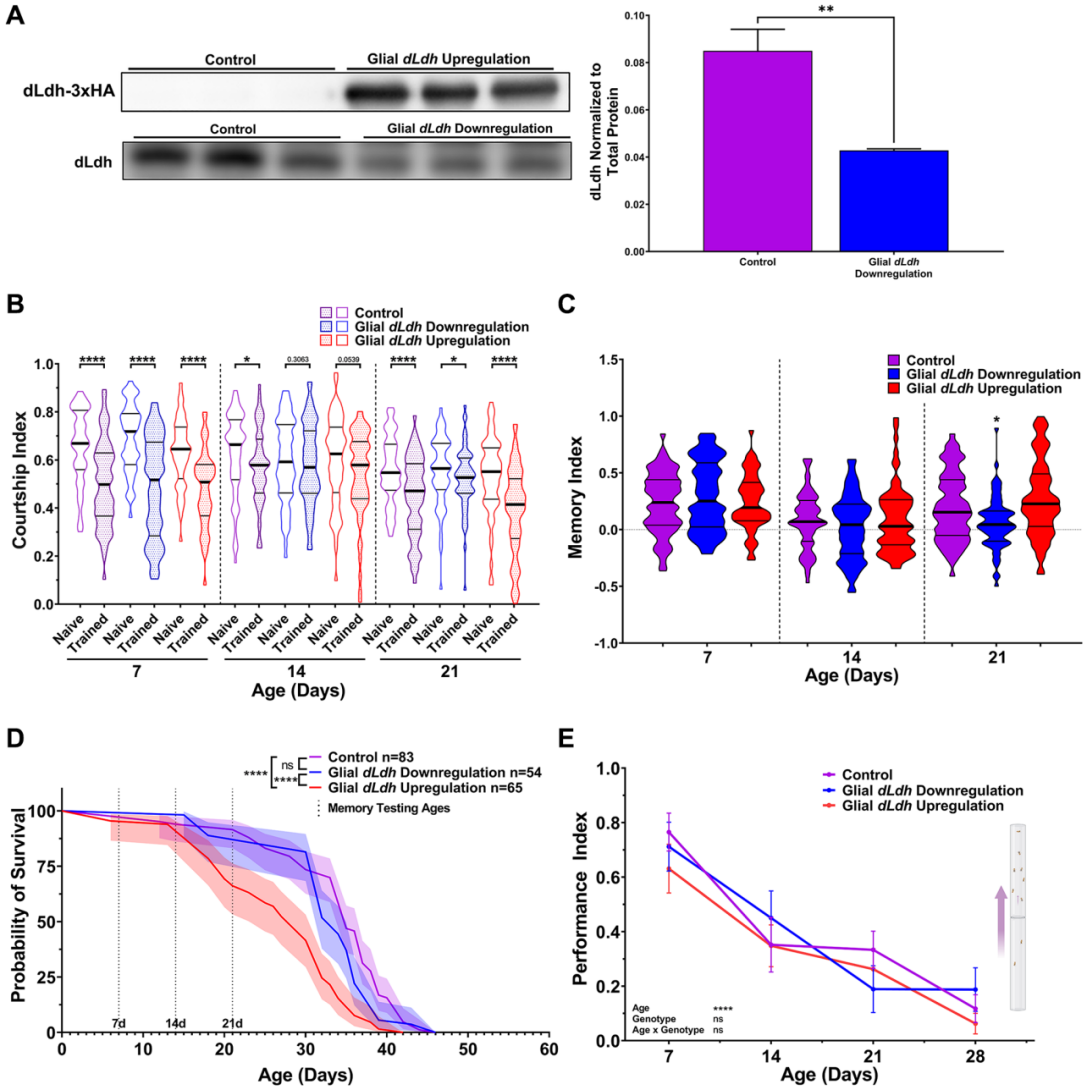
**Male flies with downregulated *dLdh* expression in glia exhibit decreased long-term courtship memory with age, while flies with upregulated *dLdh* expression in glia exhibit decreased survival.** (**A**) Western blot analysis of head extracts from glial transgenic male flies aged 21 days at 29°C showing elevated ectopic HA tagged dLdh expression (upper panel) and decreased endogenous dLdh levels (lower panel) in flies using a Repots-Gal4 driver to drive UAS-dLDH (with 3xHA tag) and UAS-dLdh-RNAi expression respectively (*n* = 3), expression was normalized to total protein content (based on Ponceau-S staining) *n* = 3. Each sample consisted of head extracts from 20 heads. Comparisons made by unpaired *t* test, ^**^p < 0.01. (**B**) Courtship indexes for glial transgenic male flies aged 7, 14, or 21 days at 29°C post-eclosion showing courtship conditioning rejection training was decreased compared to naïve flies for all genotypes at 7 and 21 days of age, and control only at 14 days of age. *n* = 48–72. Naïve and trained flies were compared within each genotype at each age using one-sided Mann-Whitney *U* tests, ^****^p < 0.0001, ^*^p < 0.05. Data presented as violin plot of frequency distribution. (**C**) Long-term courtship memory indexes for glial transgenic trained flies represented in B. Memory was only decreased following glial *dLdh* downregulation at 21 days of age compared to control (*H* = 16.9*, p* = 0.0002). Genotypes at each age were compared using Kruskal-Wallis tests with Dunn’s multiple comparisons to control, ^*^p < 0.05. Data presented as violin plot of frequency distribution. (**D**) In male flies, only glial upregulation of *dLdh* reduced survival compared to control and *dLdh* downregulation. Shaded area represents the 95% confidence interval. Curve comparison was made using a Log-rank (Mantel-Cox) test, ^****^p < 0.0001. Ages chosen for memory testing are highlighted with vertical dotted lines. (**E**) Altered glial *dLdh* expression did not impact climbing ability in male flies during negative geotaxis at any age compared to control. Comparisons across age and genotype were made using a mixed-effects model with Geisser-Greenhouse correction and Tukey’s multiple comparisons within each age group. Effects of age, genotype, and age by genotype interaction are denoted on the bottom left.

To restrict manipulation of *dLdh* expression to glial cells in adults, we employed flies containing a pan-glial expressing Repo driver combined with the same temperature system used for manipulation of neuronal *dLdh* expression (Repots-Gal4). We verified decreased dLdh protein levels in the heads of glial *dLdh* downregulated flies and induced HA-tagged dLdh levels in the heads of glial *dLdh* upregulated flies at 21 days of age by western blot analysis (Figure 3A, Supplementary Figures 3 and 4). We used courtship conditioning to assess memory in these flies. Glial *dLdh* transgenic flies exhibited no significant differences in memory compared to control flies at 7 and 14 days of age (Figure 3B and 3C), indicating that long-term courtship memory was unimpacted by glial *dLdh* manipulation at these ages. In contrast, a clear deficit in long-term courtship memory was observed in flies with glial *dLdh* downregulation compared to control flies at 21 days of age (Figure 3B and 3C). Glial *dLdh* upregulation had no effect on memory at 21 days of age (Figure 3B and 3C). The effect of altered glial *dLdh* expression on lifespan was also assessed. We detected a reduction in the survival of flies with glial *dLdh* upregulation compared to control flies, with an earlier onset of death, a slight increase in the rate of death, and minimal change in maximum lifespan (Figure 3D). Flies with glial *dLdh* downregulation had indistinguishable survival compared to control flies (Figure 3D). The climbing ability of flies with either glial *dLdh* upregulation or downregulation did not differ from control flies at all ages (Figure 3E).

### Elevated *dLdh* expression in either neurons or glia promotes neutral lipid accumulation in the brains of aged flies

**Figure 4:**
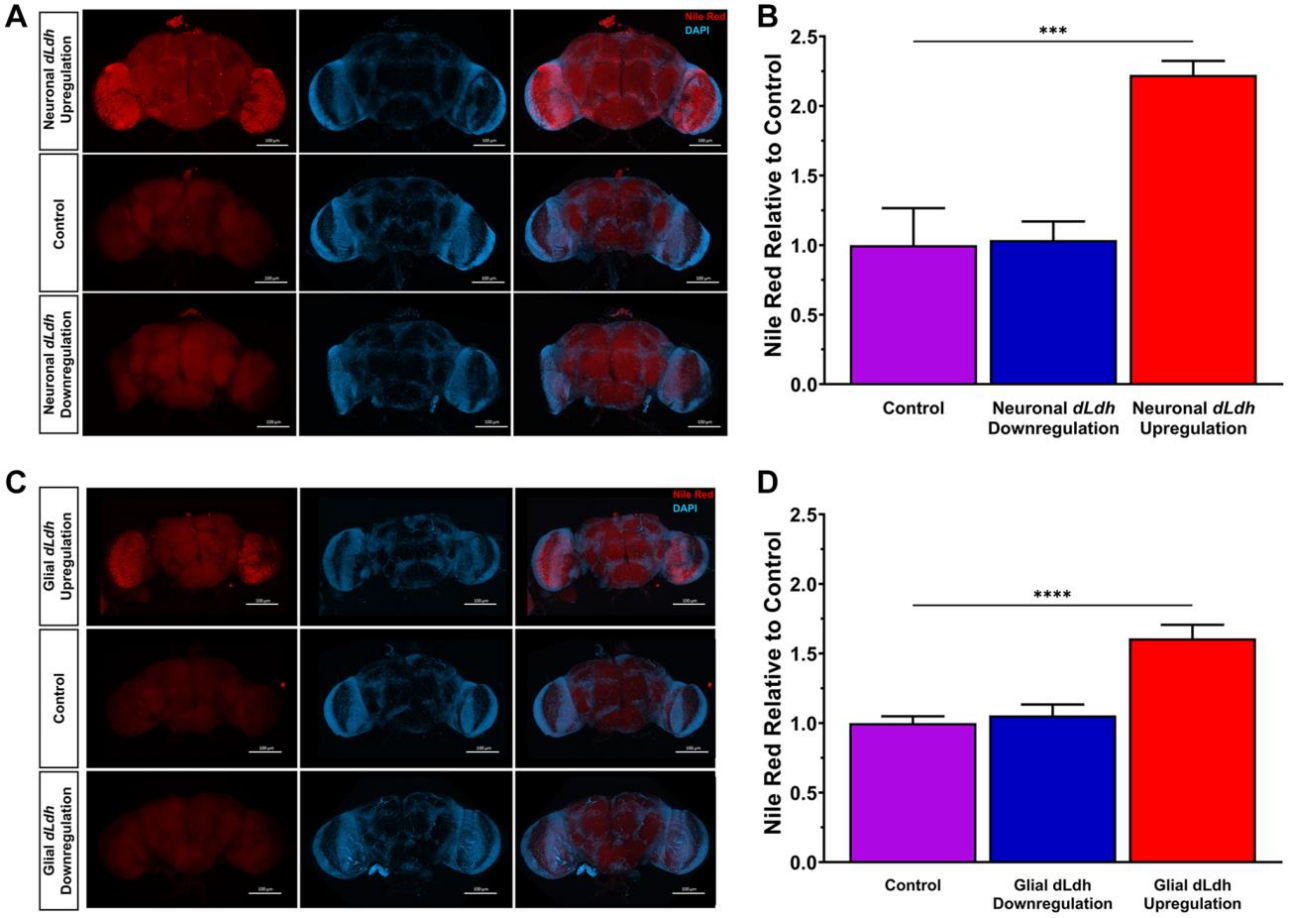
**Increased glial or neuronal *dLdh* expression promotes accumulation of neutral lipids in 21 day aged male transgenic fly whole brains.** (**A**) Representative confocal fluorescence microscopy images of neuronal *dLdh* transgenic male fly brains removed at 21 days of age at 29°C and stained for DNA (Blue = DAPI) and neutral lipids (Red = Nile Red). (**B**) Quantification of nile red staining mean fluorescence intensity revealed that male flies with neuronal *dLdh* upregulation have over twice the lipid accumulation as control flies. N = 4–5 (*F*(2, 10) = 17.69, *p* = 0.0005). (**C**) Representative confocal fluorescence microscopy images of glial *dLdh* transgenic male fly brains dissected out at 21 days of age and stained for DNA (Blue = DAPI) and neutral lipids (Red = Nile Red). (**D**) Quantification of nile red staining mean fluorescence intensity revealed that male flies with glial *dLdh* upregulation have over 1.5 times lipid accumulation as control flies. *N* = 7–8. (*F*(2, 19) = 19.74, *p* < 0.0001). Comparisons between genotypes for neuronal and glial *dLdh* transgenic flies separately were made using one-way ANOVA with Dunnett’s multiple comparisons with control. ^***^p < 0.001, ^****^p < 0.0001.

Glial generated lactate is a substrate for neuronal lipid synthesis [41]. Therefore, we investigated whether lipids were altered in the brains of aged transgenic flies in which either neuronal or glial lactate metabolism was genetically manipulated. We used nile red staining on whole brains dissected from flies at 21 days of age to detect neutral lipids [97, 98]. Brains of flies with *dLdh* upregulated in either neurons or in glia displayed a pronounced increase in neutral lipids compared to control flies (Figure 4, Supplementary Figure 5). Downregulation of *dLdh* in either neurons or glia had no apparent impact on neutral lipids detected in the brain, as assessed by nile red staining (Figure 4, Supplementary Figure 5).

### Metabolite levels in the heads of flies fluctuate with age and are readily altered by manipulating glial or neuronal lactate dehydrogenase expression

**Figure 5:**
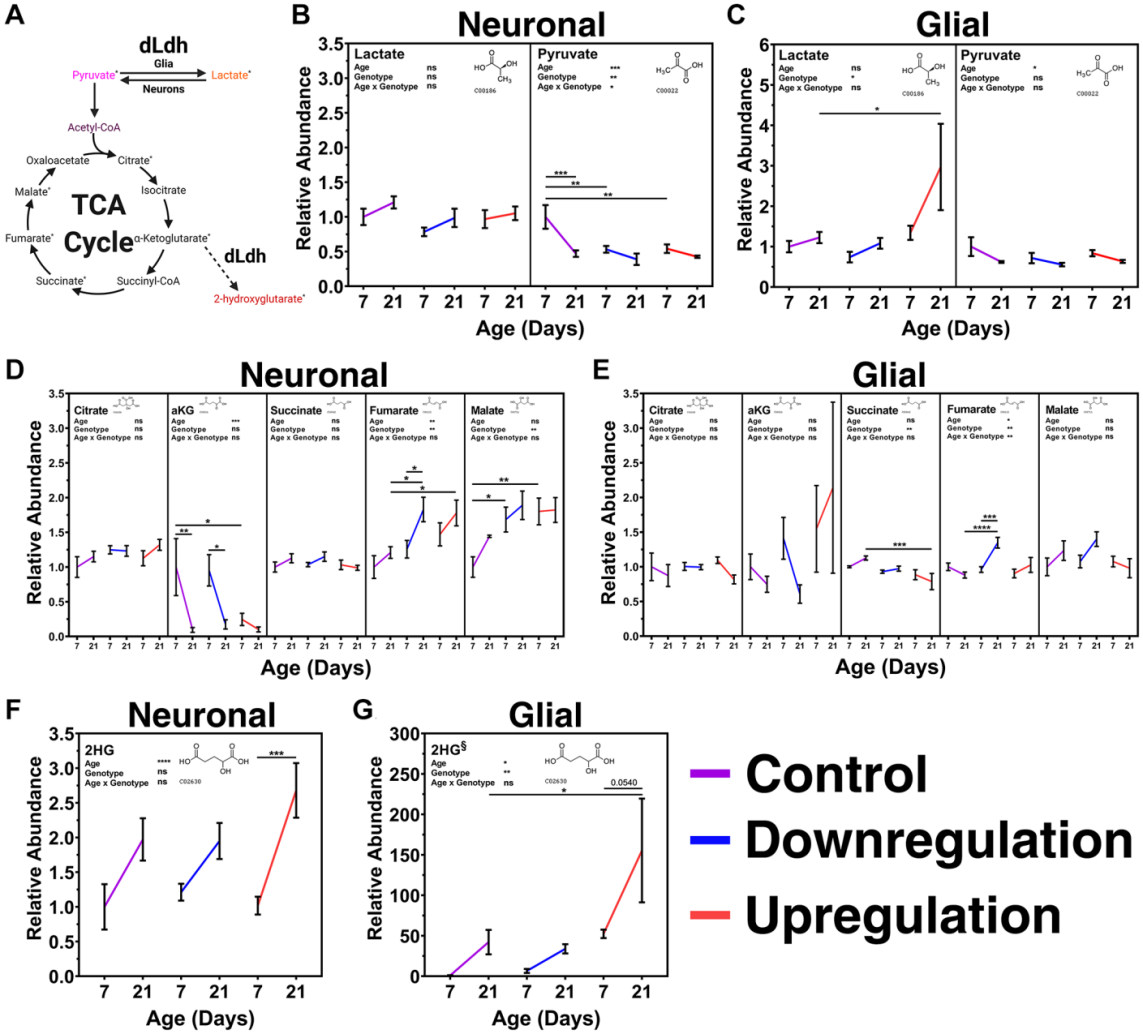
**Metabolite analysis of transgenic male fly heads aged 7 and 21 days with altered expression of neuronal or glial *dLdh* revealed alterations in lactate, pyruvate, TCA cycle intermediates, and 2HG levels relative to control flies.** (**A**) Metabolic pathway connections with metabolites produced by dLdh canonical [Pyruvate ↔ Lactate] and non-canonical [αKG→2HG] activity. Metabolites measured by gas chromatography-mass spectrometry (GC-MS) in the heads of transgenic male flies aged 7 and 21 days at 29°C are denoted by a superscript asterisk. (**B**) Neuronal *dLdh* manipulation does not impact lactate levels whereas pyruvate levels are reduced at 7 days of age nearly to the level of 21 days of age by both upregulation and downregulation. (**C**) Glial *dLdh* upregulation caused an increase in lactate but no effect on pyruvate levels. (**D**) Neuronal *dLdh* manipulation promotes alterations in select TCA cycle intermediates. Upregulation of *dLdh* lowered α-ketoglutarate (αKG) levels. Downregulation and upregulation of *dLdh* raised fumarate and malate levels. (**E**) Glial *dLdh* manipulation caused only slight alterations in TCA cycle intermediates. Citrate, αKG, and malate levels were unaltered. Upregulation of *dLdh* lowered succinate levels. Downregulation of *dLdh* raised fumarate levels at 21 days of age. (**F**) 2-hydroxyglutarate (2HG) levels are generally increased with age without any clear change due to neuronal dLdh manipulation. (**G**) The age-related increase in 2HG is exacerbated by glial *dLdh* upregulation but not by downregulation. Comparisons for each metabolite were done between genotype and age groups of neuronal and glial *dLdh* transgenic flies separately using two-way ANOVAs with Dunnet’s multiple comparisons with control for each age group and with Šídák’s multiple comparisons between age groups within each genotype. Effects of age, genotype, and age by genotype interaction are denoted on the top left of each graph. The structural formula for each metabolite were obtained from the Kyoto Encyclopedia of Genes and Genomes (KEGG) chemical compound database with associated C number below.

To determine how manipulation of *dLdh* in neurons or glia impact age-related changes in metabolism across the brain, we assessed metabolites extracted from transgenic fly heads at 7 and 21 days of age using gas chromatography-mass spectrometry (Figure 5, Supplementary Figure 6). We assumed differences in metabolite levels in transgenic fly head extracts were primarily due to changes in the brain because other structures in the head, such as cuticle and fat bodies, are unaffected by the genetic manipulation we employed to alter lactate metabolism exclusively in CNS cell types. In our metabolite analysis, we initially focused on lactate and pyruvate as these are the metabolites that are directly impacted by dLdh canonical activity (Figure 5A). Surprisingly, brain levels of lactate were unaffected by neuronal *dLdh* manipulation (Figure 5B). However, the age-related decline in brain pyruvate evident in control flies was prematurely triggered by both downregulation and upregulation of neuronal *dLdh,* with a clear reduction at 7 days of age that was maintained until 21 days of age in neuronal transgenic flies (Figure 5B). Glial *dLdh* upregulation caused a pronounced accumulation of lactate at 21 days of age, whereas downregulation had no impact on overall brain lactate levels (Figure 5C). The age-related decline in brain pyruvate levels was unaffected by both glial *dLdh* upregulation and downregulation (Figure 5C). Next, we examined the effects of altered *dLdh* expression on mitochondrial metabolism by assessing changes in TCA cycle intermediates, including citrate, alpha-ketoglutarate (αKG), succinate, fumarate, and malate (Figure 5A). We saw no differences in brain citrate level regardless of age or *dLdh* manipulation (Figure 5D and 5E). In control flies for neuronal *dLdh* manipulation, brain levels of αKG showed a substantial decline at 21 days of age (Figure 5D), whereas in neuronal *dLdh* upregulation flies, low levels of αKG in the brain were detected at both 7 and 21 days of age (Figure 5D). In both the glial *dLdh* upregulation and downregulation lines there were no changes in brain αKG levels at either 7 or 21 days of age (Figure 5E). A small decrease in brain succinate levels was observed in flies with glial *dLdh* upregulation compared to the control flies, particularly at 21 days of age (Figure 5E). Both upregulation and downregulation of neuronal *dLdh* promoted an age-related increase in brain fumarate levels compared with the control flies (Figure 5D). An age-related elevation in brain fumarate was only evident in flies with glial *dLdh* downregulation but not upregulation (Figure 5E). Malate levels in the brain were increased in both neuronal *dLdh* upregulated and downregulated flies, but this difference was most pronounced at 7 days of age relative to control flies (Figure 5D). Glial *dLdh* manipulation had no effect on malate levels at any age (Figure 5E). Lastly, we measured brain levels of 2-hydroxyglutarate (2HG), which consists of both the D- and L- enantiomers (Figure 5A). In both control and neuronal *dLdh* transgenic flies we detected increased 2HG levels in brains with age. (Figure 5F). Interestingly, we found an increase in 2HG levels in flies with glial *dLdh* upregulation compared with control, with approximately 155-fold higher 2HG levels at 21 days of age compared to 7-day old control flies (Figure 5G). Considering that dLdh catalyzes the formation of L-2HG from αKG, this pronounced increase in 2HG level likely represents increased production of L-2HG.

## DISCUSSION

### *dLdh* expression needs to be precisely regulated to ensure optimal aging

Here we show that downregulation of *dLdh* in neurons, but not glia, leads to lower survival. In accordance with glia-neuron lactate shuttling, the lower survival caused by neuronal downregulation of *dLdh* may be attributed to the decreased ability of neurons to oxidize glial-derived lactate to pyruvate in order to fuel synaptic activity and neuronal maintenance. Indeed, we detected decreased pyruvate levels in neuronal dLdh downregulated flies at 7 days of age compared to control flies. A previous study demonstrated that suppressing glycolytic enzyme expression in glia resulted in the decreased ability of glia to process trehalose as a fuel source, with a corresponding reduction in survival [39]. We previously demonstrated that glial *dLdh* downregulation in group housed flies led to increased survival [45]. However, in the current study, we detected no change in survival in males with glial *dLdh* downregulation, reared in isolation. Social isolation is known to impact aging, as well as many physiological processes [99–101]. It is possible that interfering with the ability of neurons to oxidize lactate in neuronal *dLdh* downregulation flies prevents neurons from freely shifting their metabolism in an adaptive manner to accommodate age-related changes in nutrient availability and mitochondrial oxidative capacity. In contrast, decreased glial-generated lactate in glial *dLdh* downregulation flies still allows neurons to utilize cell-autonomously generated lactate or to adapt by shifting metabolism towards utilization of other types of fuel sources or employing alternative metabolic pathways.

The question arises as to why survival was not decreased by glial *dLdh* downregulation in a manner similar to that observed following downregulation of other glycolytic enzymes [39]? We propose that a lack of glial dLdh-derived lactate for fueling neurons could be balanced by preventing damage from excess dLdh activity arising with age. The adverse effects elicited by elevated dLdh activity are exemplified by our results here showing that increasing *dLdh* in either glia or neurons causes decreased survival in *D. melanogaster.* In a previous study we found that neuronal *dLdh* upregulation caused an increase in the number of brain vacuoles [45], indicative of neurodegeneration occurring in the fly brain with age [102]. Other studies in flies have recently shown that the toxic effect of amyloid beta, a peptide strongly implicated in neurodegeneration in Alzheimer’s disease, causes *dLdh* upregulation in part due to an Activating transcription factor 4 (ATF-4) dependent endoplasmic reticulum stress signalling unfolded protein response [44]. In accordance with our findings on neuronal dLdh impact on fly survival, the authors of this study also found that both upregulation and downregulation of neuronal *dLdh* caused an exacerbation of amyloid beta toxicity [44]. Furthermore, a recent finding demonstrated that ubiquitous disruption of the *Ldhb* gene within mice resulted in increased mitochondrial dysfunction, oxidative stress, apoptosis, and cognitive impairment [103]. However, this study did not distinguish which cell type was adversely affected nor did it assess the effects of reduced *Ldhb* expression on global metabolism and brain health with age. It will be essential for future studies to determine if altered lactate metabolism in flies promotes neuronal loss in a cell-type specific manner with age.

### *dLdh* impacts age-related changes in brain metabolism differentially in neurons and glia

Aging in flies is associated with higher TCA cycle activity and nicotinamide adenine dinucleotide + (NAD+) consumption [104], in addition to a decline in mitochondrial electron transport chain (ETC) activity [79, 105, 106]. One possible explanation for decreased survival in neuronal *dLdh* overexpression is that chronic lactate oxidation in neurons may have triggered an age-related shift in neuronal metabolism towards accelerated TCA cycle activity at a younger age. The metabolic profile of neuronal *dLdh* transgenic flies in this study supports this claim. Pyruvate feeds the TCA cycle following conversion to acetyl-CoA and the subsequent formation of a succession of intermediates including citrate, isocitrate, αKG, succinyl-CoA, succinate, fumarate, malate, and ends with oxaloacetate that can react with a new acetyl-CoA to start the cycle again. We found a premature decrease in pyruvate in the heads of neuronal *dLdh* upregulated flies which may have arisen due to neurons processing pyruvate more readily through the TCA cycle at an earlier age. Moreover, in neuronal *dLdh* upregulation fly heads we saw a premature decrease in αKG, which is produced earlier in the TCA cycle after entry of pyruvate via acetyl-CoA, along with a premature increase in fumarate and malate, which are metabolites later in the TCA cycle relative to pyruvate. These changes in TCA cycle intermediates could be an indication of higher early TCA cycle activity but incomplete processing of later TCA products to renew the cycle in flies with neuronal *dLdh* upregulation.

Similarly in neuronal *dLdh* downregulated flies we saw a premature decrease in pyruvate and an increase in TCA cycle intermediates further downstream of pyruvate, including fumarate and malate, yet αKG was unchanged. If neurons with downregulated *dLdh* have reduced ability to oxidize lactate, then an increased TCA cycle activity in neurons could be due to increased utilization of non-lactate derived pyruvate sources or an increase in metabolites entering the TCA cycle through anaplerotic reactions. For example, serine is an amino acid which can function as a source of pyruvate when acted on by enzymes with L-serine ammonia-lyase activity. A gene (FBgn0037684) was recently identified in the *D. melanogaster* brain that encodes for a protein predicted to have this function [107, 108]. We detected a particularly high level of serine in the heads of flies with neuronal *dLdh* downregulation or glial *dLdh* upregulation (Supplementary Figure 6). Studies in mice suggest serine can contribute to astrocyte metabolism [109], memory loss in Alzheimer’s disease [110], oxidative stress response in T cells [111], and fuel for one-carbon metabolism [112, 113]. Moreover, D-serine is a co-agonist of N-methyl-D-aspartate (NMDA) receptors that is produced by both neurons and glia [114] and is involved in age-related memory impairment of short-term aversive olfactory associative memory in flies [59]. Therefore, further experiments are required to elucidate how the role of serine is involved in the impact of lactate metabolism on the aging brain.

Elevated fumarate levels were detected in the heads of both neuronal *dLdh* upregulation and downregulation flies which could be due to either elevated succinate dehydrogenase (SDH) or decreased fumarase activity in the TCA cycle. SDH is the only complex in the ETC which is not encoded by genes in the mitochondrial genome. Therefore, SDH function may be less impacted with age than other ETC complexes because the genes encoding the SDH complex are not as susceptible to age-related increases in oxidative damage compared to mitochondrial genes [115–117]. Overall, our metabolite analysis suggests that *D. melanogaster* neurons require finely regulated expression of *dLdh* to cope with age-related brain metabolic changes, such as high TCA cycle activity and mitochondrial dysfunction, to ensure optimal lifespan. Because there are many complex and overlapping metabolic pathways that intersect with pyruvate metabolism and the TCA cycle [118], changes in TCA cycle intermediates are not always congruent with overall TCA cycle activity and mitochondrial function. Further studies in which each TCA cycle enzyme is altered separately or in conjunction with *dLdh* manipulation are required to identify any individual TCA cycle intermediate that may participate in age-related changes either alone or in combination with altered *dLdh* expression.

Interestingly, many metabolites were altered in the same direction when *dLdh* was either overexpressed or downregulated. One possible interpretation of these findings is that *dLdh*-mediated alterations to lactate/pyruvate metabolism may elicit compensatory mechanisms to maintain metabolic homeostasis, particularly in young flies. However, many of the changes in metabolite levels in *dLdh* transgenic flies, such as lactate, fumarate, 2HG, serine, and tyrosine, only became significant at 21 days of age. Thus, it is possible that with age animals are less able to adapt or compensate for altered lactate/pyruvate levels.

Although flies with both glial and neuronal dLdh upregulation exhibited shortened lifespans, the survival curves for both lines revealed subtle differences. While neuronal and glial *dLdh* upregulated flies had an earlier onset of death, only neuronal *dLdh* upregulated flies had a reduced maximum lifespan. In addition, neuronal *dLdh* upregulated flies were the only *dLdh* manipulated group which showed a decrease in climbing ability compared to control flies, indicative of a lower healthspan [119]. The decrease in maximum longevity in flies with upregulated neuronal *dLdh* may be attributed to an earlier onset of dying with no apparent effect on the mortality rate, relative to the control lines. In contrast, a similar early onset of dying in flies with glial *dLdh* upregulation had no effect on maximum longevity likely due to the slower mortality rate in these flies relative to the control line. Therefore, an age-related increase in neuronal TCA cycle activity may not necessarily explain these glial *dLdh* induced survival deficits. This is corroborated by our observation that TCA cycle intermediates were mostly unchanged in the heads of glial *dLdh* transgenic flies. The only change in TCA cycle intermediates that we saw in flies with glial *dLdh* upregulation was a slight drop in succinate. Interestingly, we detected an accumulation of lactate only in the heads of flies with glial but not neuronal *dLdh* upregulation. Because we were only able to detect pyruvate changes in heads of neuronal transgenic flies and lactate changes in heads of glial transgenic flies, these observations suggests that in *D. melanogaster* heads it is likely that neurons play a stronger role in regulating pyruvate levels and glia are more responsible for regulating lactate levels. Moreover, *D. melanogaster* neurons may lack the ability to release lactate, unlike glia [39]. Therefore, glia with *dLdh* upregulation may produce excess lactate above the capacity of neurons to take up and oxidize this metabolite, causing lactate to accumulate to detrimental levels. Overall, our metabolite analysis suggests that decreased survival in glial *dLdh* upregulation files cannot be fully explained by age-related changes in TCA cycle intermediates, but glial overproduction of lactate may be a contributing factor.

### L-2HG may be a novel biomarker of brain aging which is increased by glial *dLdh*

Another metabolite which could be relevant to survival deficits caused by altered *dLdh* expression and aging is L-2HG. In the heads of flies with altered neuronal *dLdh* expression and their respective genotype control, we found a sharp increase in 2HG with age. Interestingly, we found an even larger increase of 2HG with age in the heads of flies with *dLdh* expression altered in glia and their respective genotype control; with 2HG elevated 155-fold by *dLdh* upregulation compared to control. L-2HG is a common product of Dipteran larval metabolism [120]. L-2HG accumulates in *D. melanogaster* larvae due to non-canonical activity of dLdh leading to the conversion of αKG to L-2HG [121], or by inhibition the enzyme L-2-hydroxyglutarate dehydrogenase (dL2HGDH) that degrades L-2HG [121]. However, L-2HG levels in *D. melanogaster* are much lower in adults compared to larvae [121] and a recent study searching for biomarkers of aging in *D. melanogaster* failed to measure 2HG [122]. To our knowledge, we present here the first demonstration that 2HG increases in the brain of flies with age. One caveat with these findings is that our method of 2HG detection included both L-2HG and D-2HG enantiomers. Although D- and L-2HG are present at similar levels in adults [120], the two enantiomers are produced by distinct enzymatic mechanisms. Since *dLdh* expression is elevated in aging brains and glial *dLdh* upregulation drives a dramatic increase in 2HG levels, our findings are likely attributable to L-2HG accumulation. In the past, L-2HG has only been found to increase to appreciable levels in the adult brain of *D. melanogaster* through an ATF4-dependent unfolded protein response when mitochondrial stress is induced [123], or in flies exposed to hypoxic conditions [120]. Therefore, aging may cause accumulation of L-2HG due to a change in brain metabolism reminiscent of responses to mitochondrial stress or hypoxia, and these metabolic pathways may be further activated in the brains of glial *dLdh* upregulation flies due to elevated lactate accumulation. Elevated levels of L-2HG in older flies may arise from lactate-mediated inhibition of L-2HG degradation in addition to non-canonical dLdh activity generating L-2HG. While L-2HG may serve as a biomarker of an age-related shift in brain metabolism, elevated L-2HG levels may directly cause damage to the brain as well. L-2HG can directly inhibit ATP synthase (Complex I in the mitochondrial ETC) [124] and αKG dehydrogenase [125], provoke oxidative DNA damage [126], and cause severe neurological problems when it accumulates in children with inborn 2-hydroxyglutaric acidurias [127] or late-onset neurodegeneration when it accumulates in mice [128]. In fact, L-2HG production in response to age-related brain metabolic changes may be a general feature of brain aging and may be partially responsible for decreased survival caused by glial *dLdh* upregulation and lactate accumulation.

### Accumulation of neutral lipids in the brain may underlie age-related impacts of *dLdh*

There is an increasing recognition of the role that lipid metabolism plays in aging [129]. Lipids are typically stored as triglycerides and sterol esters in lipid droplet intracellular organelles for energy production [130, 131]. Neutral lipids are covered in a monolayer of phospholipids and proteins in lipid droplets and can be broken down into fatty acid and cholesterol by lipolysis or autophagy (lipophagy) [132, 133]. Here we show a marked increase in neutral lipids in the brain of aged flies with *dLdh* upregulated in either glia or neurons, suggesting lipid droplet accumulation in both cases. Accumulation of lipid droplets is known to occur in the brain during development [134, 135], upon aging [136–138] and in response to stressors such as mitochondrial dysfunction [41, 139], oxidative stress [97, 139–141], ER stress [142, 143], inflammation [138], starvation [97, 143], and neurodegenerative disease [144–147]. Outside of lipid energy storage, there are a variety of potential lipid droplet functions, including protein maturation and turnover, intracellular motility, transcriptional regulation in the nucleus, and storage of proteins, vitamins, and signaling precursors [130]. In the brain, glia form lipid droplets more readily than neurons [132]. *D. melanogaster* larval brains accumulate lipid droplets exclusively in glia [134], whereas adulthood lipid droplets are predominantly localized in glia [97, 140] and to a lesser extent in neurons [148, 149]. Neurons retain less lipid droplets than glia normally to avoid lipotoxicity occurring during the production of reactive oxygen species from free fatty acid β-oxidation [150, 151]. Interestingly, it has been shown in mice and flies that the glia-neuron lactate shuttle provides a neuronal substrate for synthesis of fatty acids, but neurons avoid fatty acid lipotoxicity by shuttling fatty acids via apolipoproteins back to glia for incorporation in lipid droplets [41, 139, 146, 152–154]. The trading of lactate for fatty acids between glia and neurons to trigger glial lipid droplet production was shown to occur in the *D. melanogaster* retina and was prevented by knockdown of *dLdh* in either all neurons or pigment glia residing in the ommatidia [41]. Our results demonstrate that overproduction of *dLdh* in neurons or glia is sufficient to trigger neutral lipid accumulation outside of ommatidia and in the rest of the brain as well. Moreover, if neutral lipid accumulation is an indication of glial lipid droplets forming as an adaptive response to an age-related increase in neuronal mitochondrial dysfunction and oxidative stress, then elevation of these processes brain wide may partly explain why we observed reduced survival in flies with glial or neuronal *dLdh* upregulation in this study. In addition, if neutral lipid accumulation is an indication of increased neuronal lipid droplet formation, then survival deficits in flies with glial or neuronal *dLdh* upregulation could be due to a shift in neuronal metabolism towards excessive neuronal triglyceride synthesis which can trigger lipotoxicity when utilized for energy. Future investigation into the cellular distribution of lipid droplet accumulation under conditions of dLdh induced changes in aging will be highly illuminating. Overall, our results suggest aberrant lipid metabolism caused by neuronal and glial *dLdh* upregulation may be a contributing factor to decreased lifespan.

### *dLdh* involvement in long-term memory is age and cell-type dependent

In this study we show that neuronal or glial downregulation or neuronal upregulation of *dLdh* in *D. melanogaster* each cause a deficit in long-term courtship memory but only with age. On the other hand, we found glial *dLdh* upregulation had no impact on long-term courtship memory. We had expected that these interventions would modify glia-neuron lactate shuttling and alter memory in a manner similar to that observed following disruption to the ANLS in vertebrates such as mice, rats, and chicken [46–54, 155]. Only one study has examined the impact of the ANLS on cognition with age. That study measured the indirect involvement of the ANLS in memory by knocking out β2-adrenergic receptors in mouse astrocytes to prevent epinephrine signaling induced lactate release [156]. β2-adrenergic receptor astrocyte knockout mice had no change in memory, assessed by the Morris water maze, at a young age, but developed memory deficits at an older age [156]. Interestingly, the memory deficits we uncovered in *dLdh* transgenic flies were similarly restricted to aged animals. These findings suggest that cognitive processing in the aged brain is more susceptible to changes in either the production or shuttling of lactate between glia and neurons compared to the young brain.

In this study we used a fly behavioural paradigm testing cis-vaccenyl acetate (cVA)-retrievable courtship memory, which relies on neural circuitry that differs from associative courtship memory processing and is likely more complicated than aversive olfactory associative memory [157]. The utilization of appetitive memory as well as aversive memory neural circuits for processing of courtship memory [158] may provide redundancy in courtship memory processing. This redundancy may explain why long-term courtship memory in this study, and short-term courtship memory in previous studies [93, 94], do not exhibit age-related degradation in non-transgenic flies that is typically evident for aversive olfactory associative memory [85, 86, 159]. Moreover, we focused on long-term memory because the ANLS has been shown in mice to be dispensable for short-term memory in certain paradigms [47]. Other studies have found manipulating glia causes age-related impairment of aversive olfactory associative memory [58, 59]. We found that both glial and neuronal *dLdh* downregulation caused impairment of long-term courtship memory only in aged flies. If glial and neuronal *dLdh* downregulation reduce the ability of glia to release lactate and neurons to oxidize lactate, then these results may reflect the increased dependence on glia-neuron lactate shuttling to fuel long-term courtship memory with age. Perhaps mushroom body neurons encoding long-term courtship memory [90] increase mitochondrial energy flux, as has been proposed for long-term aversive olfactory associative memory encoding [57], but oxidation of lactate becomes a more important mitochondrial fuel supply as flies age. Moreover, an increase in glia-neuron lactate shuttling with age in mushroom bodies would explain why the levels of dLdh increase in the brain with age and why *dLdh* is enriched in mushroom bodies [77]. On the other hand, we also found overexpression of *dLdh* in neurons causes age-related impairment of long-term courtship memory. Neurons with an overabundance of dLdh should retain the ability to oxidize lactate, so a lack of lactate provided by glia seems to be an unlikely explanation for memory deficits in flies with neuronal *dLdh* upregulation. It is possible that excess lactate oxidation by dLdh in neurons promotes increased mitochondrial reactive oxygen species (ROS) production [160] and potentiates age-related neurodegeneration and memory decline. Interestingly, fumarate was the only metabolite that exhibited an elevation in levels that inversely correlated with memory in aged *dLdh* transgenic flies. The excessive buildup of fumarate in humans with Fumarate hydratase (FH) deficiency, a rare genetic disease, results in brain atrophy, neurologic abnormalities, and intellectual impairment [161, 162]. Interestingly, cancer cells with an FH deficiency produce excess fumarate that, in turn, promotes increased ROS levels (Sullivan et al., Mol Cell -2013). Whether mitochondrial lactate oxidation and/or fumarate accumulation associated with altered *dLdh* expression contributes to memory loss or ROS production awaits further investigation. Overall, our findings suggest that *D. melanogaster* long-term courtship memory maintenance with age requires dLdh levels to be tightly regulated in neurons and at or above a critical level in glia.

## CONCLUSION

**Figure 6:**
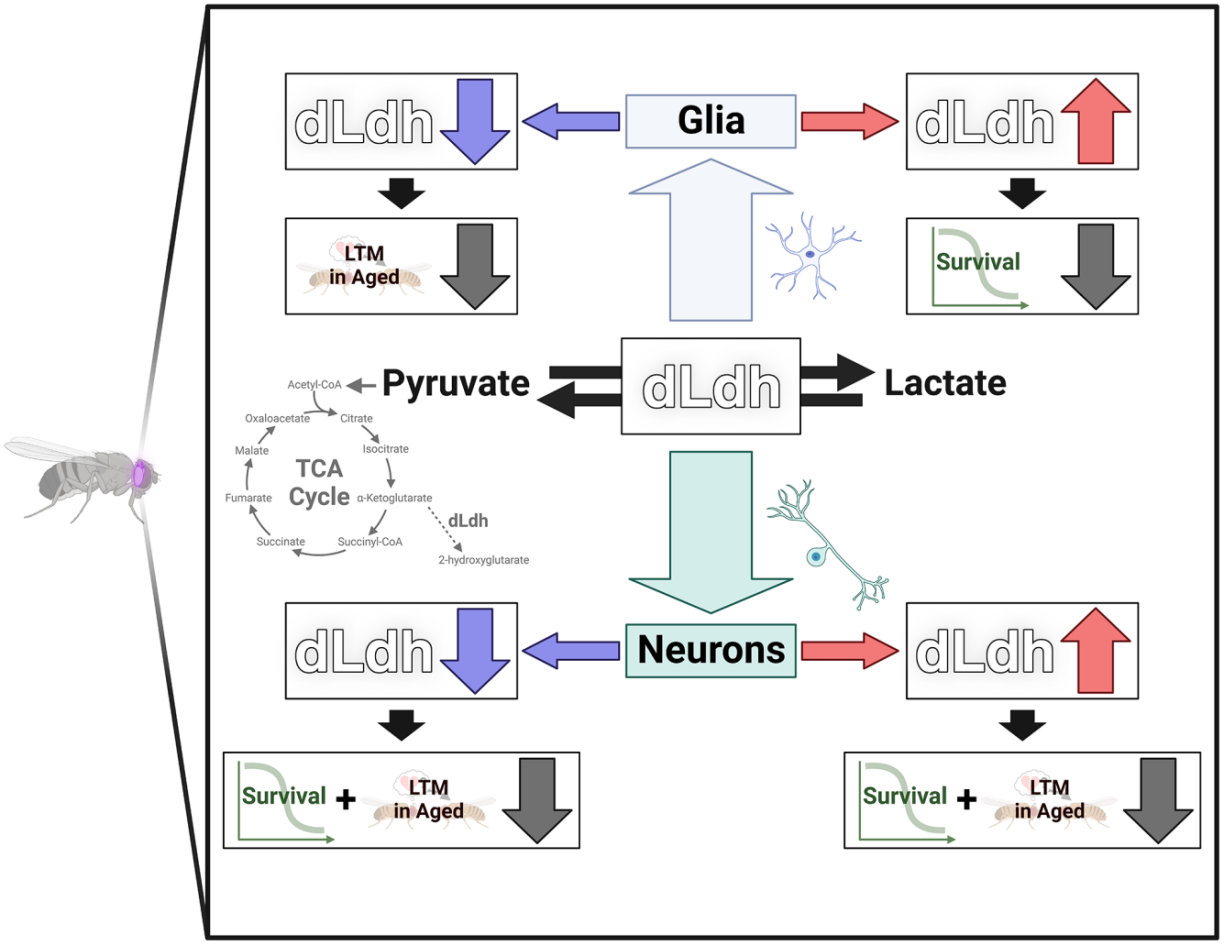
**Graphical summary dLdh level was altered in neurons or glia in the brain of flies.** Long-term courtship memory (LTM) was reduced in aged flies by increasing neuronal dLdh and decreasing neuronal or glial dLdh. Survival was reduced by increasing glial dLdh and decreasing neuronal or glial dLdh.

In this study we demonstrate the importance of maintaining appropriate levels of dLdh in *D. melanogaster* glia and neurons for maintenance of long-term courtship memory and survival with age (Figure 6). In addition, our results implicate lipid metabolism, 2HG accumulation, and changes in TCA cycle activity as factors underlying the age-related impacts of perturbed *dLdh* expression, which likely modifies glia-neuron lactate shuttling in the fly brain. Moreover, we demonstrate that normal features of *D. melanogaster* aging likely include retention of the ability to form long-term courtship memory, unlike other types of memory, and the accumulation of 2HG in the brain. This study provides a greater understanding of how aging, lactate metabolism, and cognitive processing intersect. Future work is required to determine how lactate metabolism may differ across various *D. melanogaster* brain regions with age and which subtypes of neurons and glia are most susceptible to glia-neuron lactate shuttle perturbations related to accumulation of neutral lipids and the 2HG enantiomers. Our findings highlight a connection between glia-neuron metabolic coupling and age-related memory impairment in *D. melanogaster,* a model highly amenable to further exploration of the role of metabolic coupling on memory processes with age.

## MATERIALS AND METHODS

### Key resources table

**Table:** 

**Reagent type (species) or resource**	**Designation**	**Source or reference**	**Identifier**	**Additional information**
Genetic reagent (*Drosophila melanogaster*)	Canton-S	Anne Simon’s lab		
Genetic reagent (*Drosophila melanogaster*)	Repots-Gal4	Jadwiga Giebultowicz’s lab [45]		
Genetic reagent (*Drosophila melanogaster*)	PMF	Jamie Kramer’s lab [163]		Hybrid of Canton-S and Oregon-R
Genetic reagent (*Drosophila melanogaster*)	Elavts-Gal4	Doug Allan’s lab [164]		
Genetic reagent (*Drosophila melanogaster*)	UAS-dLdh	FlyORF	F002924	
Genetic reagent (*Drosophila melanogaster*)	UAS-dLdh-RNAi	Vienna Drosophila RNAi Center	v31192	
Genetic reagent (*Drosophila melanogaster*)	*w^1118^*CS_10_	Anne Simon’s lab		*w^1118^* mutation outcrossed 10 times in our reference CS background
Commercial assay	Detergent compatible assay	Bio-Rad	5000111	
Chemical compound	Jazz-mix	ThermoFisher Scientific	AS153	Drosophila Rearing media
Chemical compound	Halt^TM^ cocktail	ThermoFisher Scientific	87786	
Chemical compound	Precision Plus Protein™ All Blue Prestained Protein Standards	Bio-Rad	1610373	
Chemical compound	Ponceau S	Millipore Sigma	P3504	
Chemical compound	Immobilon Classico or Forte Western HRP substrate	Millipore Sigma	WBLUC, WBLUF	
Chemical compound	Succinic acid-2,2,3,3-d_4_	Millipore Sigma	293075	
Chemical compound	Methoxyamine hydrochloride (MOX)	MP Biomedicals	155405	
Chemical compound	Anhydrous pyridine	Millipore Sigma	PX2012-7	
Chemical compound	N-Methyl-N-(trimethylsilyl)trifluoroacetamide (MSTFA)	Millipore Sigma	69478	
Chemical compound	Nile red	Millipore Sigma	72485	1mg/ml
Chemical compound	Vectashield mounting medium with DAPI	Vector laboratories	H-1500	
Antibody	Rabbit anti-LDHA	ThermoFisher Scientific	PA5-26531	Western Blot 1:1000
Antibody	Mouse anti-HA.11 epitope tag	BioLegend	901513	Western Blot 1:1000
Antibody	Goat anti-mouse, HRP conjugate	Millipore Sigma	AP130P	Western Blot 1:10000
Antibody	Goat anti-rabbit, HRP conjugate	Millipore Sigma	AP132P	Western Blot 1:6666
Software, algorithm	GraphPad Prism version 9.3.1 (471)	GraphPad Software	RRID:SCR_002798	
Software, algorithm	Image Lab	Bio-Rad	RRID:SCR_014210	
Software, algorithm	MassHunter Quantitative software	Agilent	RRID:SCR_015040	
Software, algorithm	ZEN Digital Imaging for Light Microscopy	Zeiss	RRID:SCR_013672	
Software, algorithm	FIJI-ImageJ	[165]	RRID:SCR_002285	
Software, algorithm	BioRender	BioRender	RRID:SCR_018361	

### Fly stocks and maintenance

*Drosophila melanogaster* fly stocks were maintained on a 12:12-h light:dark cycle at 50% relative humidity on Jazz-mix (AS153, ThermoFisher Scientific) food or food made in-house using an equivalent recipe. All experiments were done using male flies exclusively. Flies undergoing negative geotaxis assays were maintained in vials (AS507, ThermoFisher Scientific). Flies for all other experiments were maintained under individual housing conditions, each within a 2 ml deep well of a 96 well plate (89237-526, VWR) half filled with food. Flies in 96 well plates were transferred to new food at least once every two weeks. In house Canton-S flies were used as non-transgenic controls, maintained at 25°C for courtship conditioning and 25°C or 29°C for survival analysis. All transgenic flies were raised at 18°C in order to prevent activation of the temporal and regional gene expression targeting (TARGET) system then maintained at 29°C post-eclosion for experimental purposes [95]. The fly stocks used in this study were as follows: Canton-S (CS), UAS-dLdh (F002924; FlyORF), UAS-dLdh-RNAi (v31192; VDRC), Elavts-Gal4 (w,elavC155-Gal4,UAS-Dicer2;tuGal80ts,UAS-nGFP; Provided by Doug Allan, University of British Columbia), Repots-Gal4 (w; tubGal80TS;Repo-Gal4; Provided by Jaga Giebultowicz, Oregon State University), PMF (Hybrid of Canton-S and Oregon-R; Provided by Jamie Kramer, Dalhousie University), and w^1118^_CS10_. UAS-dLdh and UAS-dLdh-RNAi were outcrossed for five generations to w^1118^_CS10_, a *w^1118^* mutation outcrossed 10 times in our reference CS background.

### Survival analysis

For continuity between flies aged for courtship conditioning and flies aged for survival analysis, individual wells of 96 well plates were used for housing. Adult CS male flies were collected with or without brief CO_2_ anesthesia and maintained in 96 well plates as described above. Those flies were raised at 25°C, and their survival was assessed at 29°C or 25°C in three separate trials. For each temperature group, a survival curve was generated by consolidating data from all three trials on that group. Transgenic flies and their associated control groups lacking UAS constructs were each assessed in a trial of groups tested at 29°C, after being raised at 18°C, and all collected within a week of hatching from pupae. For all flies, the first day in 29°C conditions was considered the first day of survival. Deaths were recorded 3–4 times per week. Flies which managed to escape were censored. Due to potentially adverse health outcomes associated with social isolation and elevated temperature [101], catastrophic death events, where greater than 20% of flies suddenly died at a rate unrepresentative of the rest of the population or among repeated independent trials, were excluded [166, 167]. Catastrophic deaths were removed because they were likely non-biologically relevant deaths due to the suboptimal housing conditions of 96 well plates and not attributable to genetic predisposition to increased mortality. Survival curves were generated using percentage survival computed with the product limit (Kaplan-Meier) method for each group of flies and censored flies omitted from the graph. To compare survival curves, the log-rank (Mantel-Cox) test was used to determine differences in curves overall, median survival, maximum lifespan, and rate of dying (hazard ratio with 95% confidence interval calculated using the Mantel-Haenszel method). All calculations were done using GraphPad Prism version 9.3.1 (RRID: SCR_002798).

### Western blot

Frozen flies were decapitated and heads in a 2% sodium dodecyl sulfate (SDS) lysis buffer made with 50 mM Tris, 1mM Ethylenediaminetetraacetic acid (EDTA) and protease inhibitors, 1 mM sodium orthovanadate, 1mM phenylmethylsulfonyl fluoride, and Halt^™^ cocktail (87786, ThermoFisher Scientific). After homogenization with a pestle and sonication of the heads, the samples were centrifuged and protein in the supernatant was extracted. The concentration of extracted protein was determined using a detergent compatible assay (5000111, Bio-Rad). Extracted protein was combined with a bromophenol blue based loading buffer, boiled, resolved on an acrylamide gel alongside protein standards (1610373, Bio-Rad) and transferred to a polyvinylidene fluoride (PVDF) membrane. Total protein level was measured by staining with 0.1% Ponceau S (P3504, Millipore Sigma) in 5% glacial acetic acid for 3 minutes with excess Ponceau S removed with a 3-minute methanol wash prior to imaging. Blots were then destained, using 5-minute washes with tris buffered saline containing tween 20 (TBST) four times, and blocked with bovine serum albumin (BSA) and milk in TBST for at least 1 hour. Blots were probed with primary antibody at 4°C overnight. Primary antibodies were as follows: rabbit anti-LDHA (PA5-26531, ThermoFisher Scientific; 1:1000), and mouse anti-HA.11 epitope tag (901513, BioLegend; 1:1000). Subsequently, blots were probed with horse radish peroxidase (HRP) conjugated secondary antibodies at room temperature for 2 hours. Secondary antibodies were as follows: goat anti-mouse (AP130P, Millipore Sigma; 1:10000), and goat anti-rabbit (AP132P, Millipore Sigma; 1:6666). Antibodies were made in TBST with 0.01% sodium azide, and blots were washed in TBST before and after each probe. Immobilon Classico or Forte Western HRP substrate (WBLUC, WBLUF, Millipore Sigma) were used to detect chemiluminescent signals on probed blots. Chemiluminescence and Ponceaus S signal density were each imaged using a ChemiDoc XRS imaging system (170-8070, Bio-Rad) and quantified using Image Lab software (RRID:SCR_014210). For quantification purposes, western blot band intensity was standardized to total Ponceaus S signal density for each lane.

### Metabolite analysis using gas chromatography-mass spectrometry (GC-MS)

Frozen fly heads were stored at –80°C. Prior to analysis, heads were transferred to pre-tared 2 mL screw cap tubes containing 1.4 mm ceramic beads (15340153, ThermoFisher Scientific), the mass was recorded, and samples were placed in liquid nitrogen. As previously described [168], a solution of 90% methanol (34860, Millipore Sigma) containing a succinic acid-2,2,3,3-d_4_ (293075, Millipore Sigma) standard was added to each sample and homogenized at 4°C for 30 seconds using an Omni BeadRuptor 24 set at 6.45 m/s. The samples were incubated at −20°C for an hour, centrifuged at 10,000 × g at 4°C, and the supernatant was transferred to a 1.5 ml microfuge tube. The sample was dried overnight using a Thermo Savant vacuum centrifuge (SPD130DLX-115) set to ambient temperature and attached a Savant Refrigerated Vapor Trap (RVT5105-230). Samples were resuspended and derivatized by adding 40 μl of 40 mg/ml methoxyamine hydrochloride (MOX; 155405, MP Biomedicals) in anhydrous pyridine (PX2012-7, Millipore Sigma) and incubated at 35°C for one hour with shaking at 600 rpm using an Eppendorf ThermoMixer F1.5. Then 25 μl of the supernatant was transferred into a 2 ml sample vial containing a 250 μl deactivated glass insert (5181-8872, Agilent). 40 μl of N-Methyl-N-(trimethylsilyl)trifluoroacetamide (MSTFA; 69478, Millipore Sigma) was added and the vial placed in a Benchmark Multi-Therm heat shaker set at 40°C and 250 rpm. Following the incubation period, samples were immediately analyzed using a 7890B-5977B MSD Agilent GC-MS. 1 μl of the derivatized sample was injected into a 0.25 mm i.d., 30-meter DB-5MS column (Agilent) and the split ratio was set to 50:1. Initial oven temperature was set to 95°C with one minute of hold time. The first ramp was set to 40°C/min until 110°C, then 5°C/min until it reaches 250°C and finally 25°C/min until 330°C. The area under the peak for each metabolite was calculated using MassHunter Quantitative software (RRID: SCR_015040). The value for each metabolite was normalized to the succinic acid-2,2,3,3-d_4_ internal standard and sample mass. For each condition, outliers were identified and removed using the Robust regression and Outlier removal (ROUT) method [169]. Relative abundance of each metabolite was calculated by further normalizing to the average for control flies at 7 days of age.

### Courtship conditioning

In order to prevent male flies from having any courtship experience prior to training, they were isolated in 96 well plates either early post-eclosion, and briefly anesthetized on ice, or as pupae just prior to eclosion. Long-term courtship memory is formed by males that learn to reduce courtship to a target pre-mated female (PMF) by a training period in which the male experiences constant rejection by a PMF. PMF or CS females, aged five days in vials at 25°C in the presence of males, were collected with CO_2_ anesthesia one to five hours prior to either training or testing in order to act as the target pre-mated female. Males were trained by transferring them to a new well of a 96 well plate with a PMF for seven to eight hours. Naïve males were sham trained by transferring to a new well of a 96 well plate alone. 20–24 hours after training, long-term courtship memory testing was performed by providing a target PMF to each male in a transparent circular enclosure for 10 minutes without food. Training and testing were done under the same temperature and humidity conditions males were aged under. Video recordings of courtship during the 10 minutes testing of both naïve and trained males were generated using an iPad (A1458, Apple) or iPad mini (A1489, Apple) and scored manually, with the scorer blind to training status, genotype, and age. Male behaviours considered courtship include orienting, chasing, tapping, licking, vibrating wings, unilateral wing extension, and abdomen bending in attempt to copulate. Courtship index (CI) was calculated for each tested male as the percent of time exhibiting courtship behaviour. When CI of trained males is lower than CI of naïve males within the same age and genotype this is considered an exhibition of long-term courtship memory. Memory Indexes (MI) were calculated using CI for each trained fly compared to the average of naïve flies in the same condition [MI = 1 − (CI_Trained_/ CI_Average Naive_)].

### Negative geotaxis climbing ability assays

Male flies were raised in vials in groups of 50 with surviving flies tested weekly. Flies were transferred to new vials of food after each test to be retested the following week, up until 28 days of age. Climbing ability was assayed at 25°C and 50% humidity in a counter-current apparatus according to an adapted protocol [170, 171]. For each test, flies were transferred to a vertically oriented plastic conical vial (352017, Corning) on the bottom of the counter current device. Flies were then tapped down 6–7 times to bring them down to the bottom of the vial. With all flies on the bottom of the vial, the upper portion of the counter-current device was displaced to open access to a clean empty plastic conical vial on the top of the apparatus. Flies will climb upwards due to their natural inclination for negative geotaxis. After 10 seconds of letting flies climb upwards towards the top vial, the upper portion of the counter-current device was displaced again to separate flies that had reached the top vial from flies that had not. A performance index (PI) was calculated by dividing the number of flies which had reached the top vial within 10 seconds by the total number of flies originally placed in the bottom vial [PI = # Top Flies/Total # Flies].

### Nile red staining

Brains of male flies aged 21 days at 29°C were analyzed. Brains were dissected and stained with Nile Red for neutral lipids according to a protocol adapted from [97]. Flies were briefly anesthetized with carbon dioxide and brains dissected on ice cold phosphate buffered saline (PBS). Brains were fixed in 4% paraformaldehyde and 0.5% Triton X in PBS for 45 minutes on ice, then washed three times in PBS on ice for 15 minutes. Brains were stained with 1 μg/ml rile red in PBS on ice, freshly made from a 1 mg/ml stock of nile red (72485, Millipore Sigma) dissolved in acetone stock solution. After staining, brains were rinsed once in PBS on ice, mounted onto glass slides using Vectashield mounting medium with DAPI (H-1500, Vector Laboratories), and imaged by confocal microscopy on the same day.

### Confocal microscopy

For Nile Red staining, fly brains were imaged on an inverted Zeiss LSM 800 scanning laser confocal microscope with an LCI Plan-Neofluar 25x/0.8 Imm Korr DIC water immersion objective (Zeiss). Tiles of Z-stacks with 10 μm intervals were recorded spanning the entire brain. Nile Red and DAPI fluorescence were sequentially imaged. Nile Red fluorescence was detected using 559 μm excitation and 646 μm emission acquired with a 53 μm pinhole. DAPI fluorescence was detected using 353 μm excitation and 465 μm emission acquired with a 52 μm pinhole. Maximum intensity projections were generated for analysis and visualization (RRID: SCR_013672, ZEN Digital Imaging for Light Microscopy). Nile red mean fluorescence in the total area of each brain and in a square sample of the background was quantified in FIJI-ImageJ (Schindelin et al., 2012; RRID:SCR_002285) using the protocol described by [172]. Background was subtracted from brain signal for each brain and normalized to the average of control brains.

### Statistical analyses

Data are presented as mean ± SEM unless otherwise specified and were analyzed statistically and visualized using GraphPad Prism version 9.3.1 (471) (RRID:SCR_002798). All raw data and statistical analyses are available on the Dryad data repository (DOI:10.5061/dryad.2v6wwpzsb).

## Supplementary Materials

Supplementary Figures
