# Supplementary material for: Aging and memory are altered by genetically manipulating lactate dehydrogenase in the neurons or glia of flies
Source: Aging (Albany NY). 2023 Feb 25;15(4):947–81. doi: 10.18632/aging.204565 (PMC10008500; doi:10.18632/aging.204565)
Supplement: Supplementary Figures [file aging-15-204565-s001.pdf]

SUPPLEMENTARY FIGURES

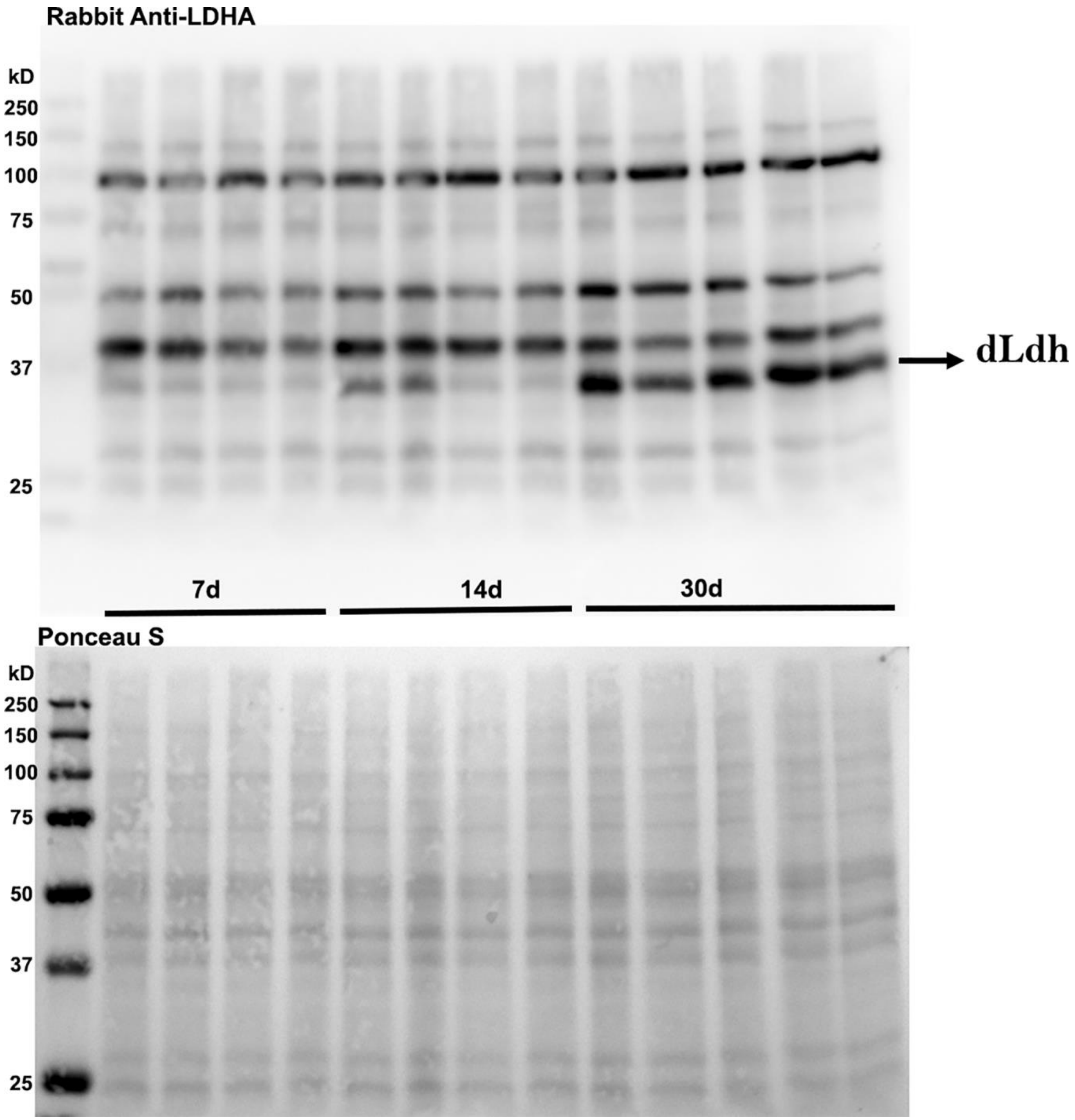

Supplementary Figure 1. Full Western blot images for Figure 1E dLdh detection. Full images for western blot analysis to detect dLdh (~35.5kD) in protein extracts from heads of male Canton-S flies aged 7, 14 or 30 days at 29°C quantified in Figure 1E. Ponceau S stained blot revealing all transferred proteins is shown below.

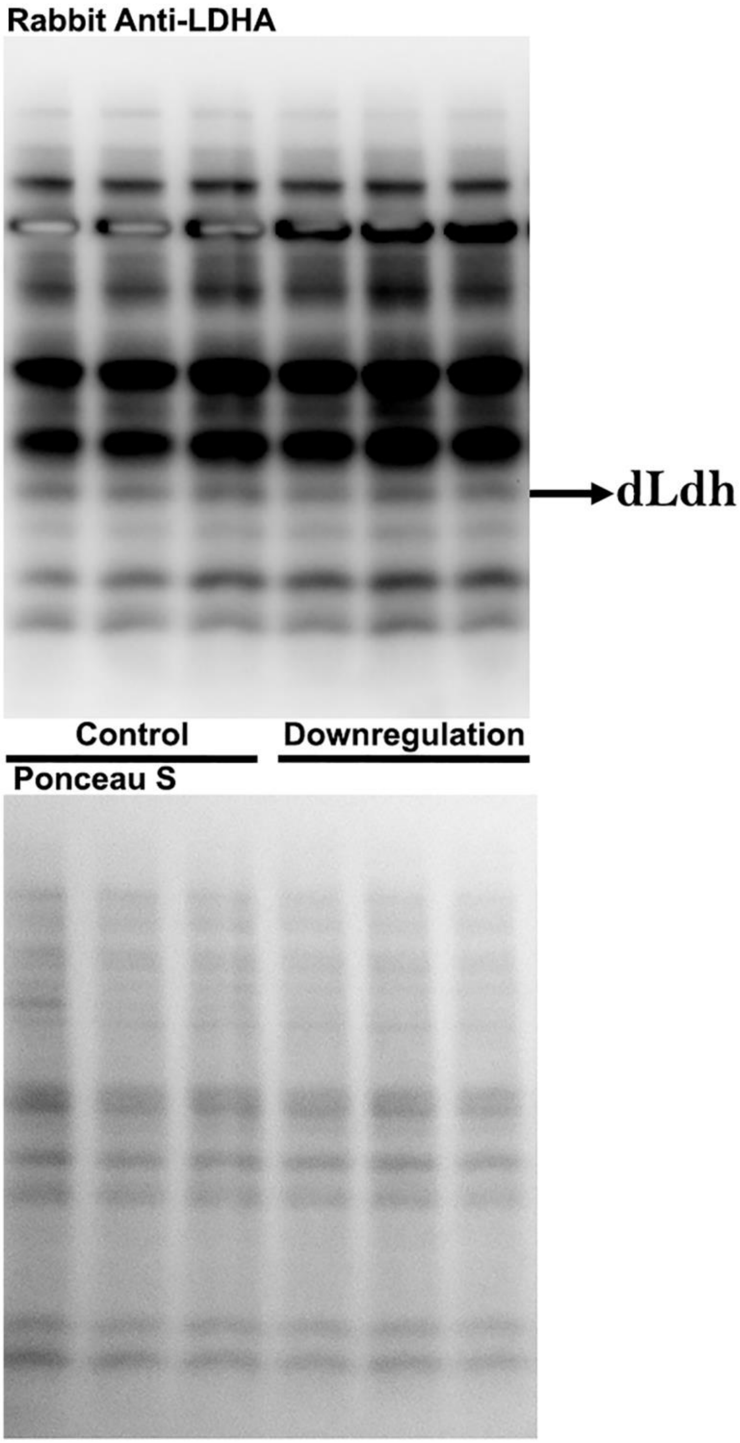

Supplementary Figure 2. Full Western blot images for Figure 2A dLdh detection. Full images for western blot analysis to detect dLdh (~35.5kD) in head extracts from neuronal transgenic male flies aged 21 days at 29°C quantified in Figure 2A. Ponceau-S stained blot revealing all transferred proteins is shown below.

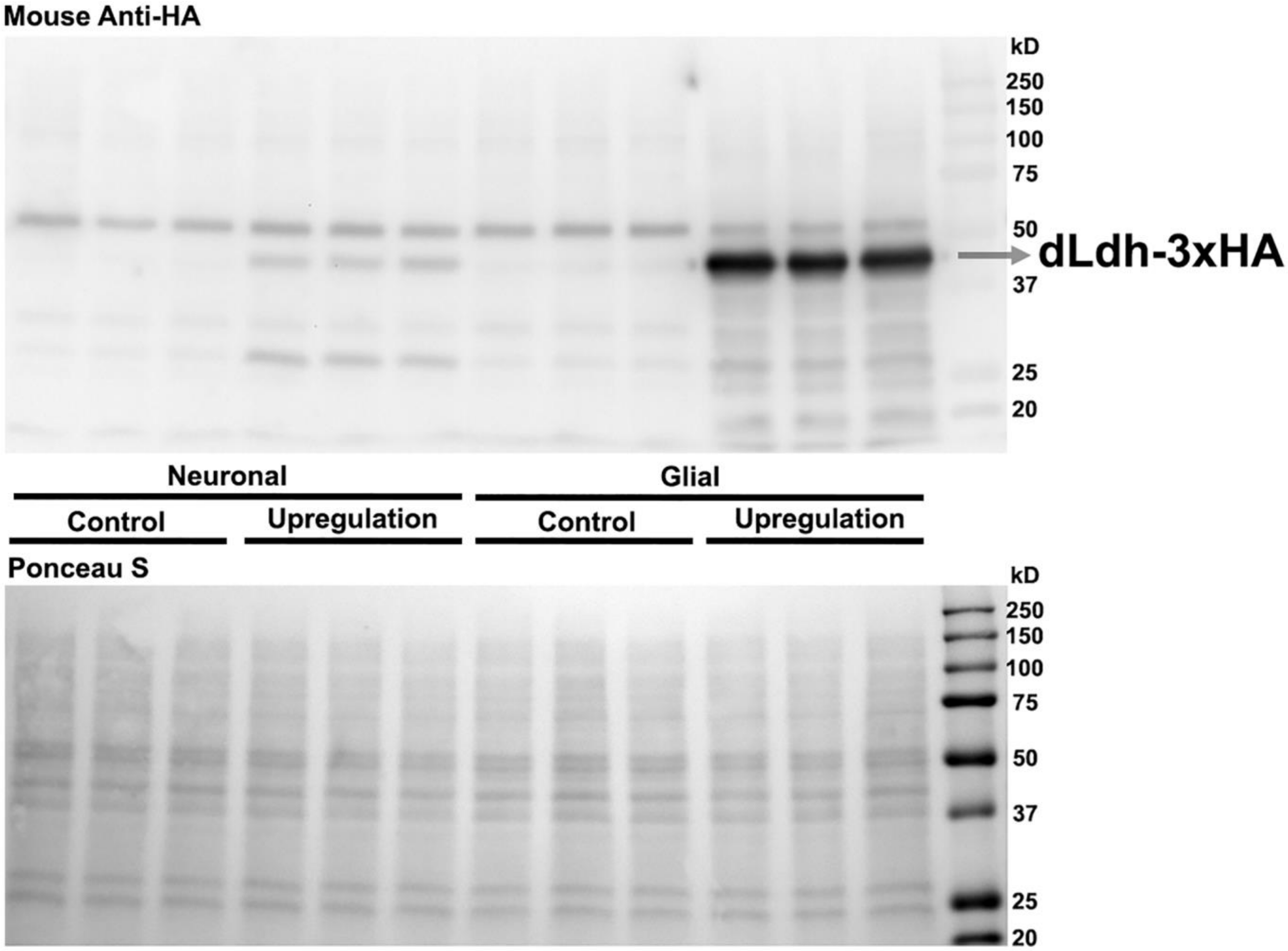

Supplementary Figure 3. Full Western blot images for Figure 2A and 3A dLdh-3xHA detection. Full images for western blot analysis to detect dLdh with a triple hemagglutinin (HA) tag (dLdh-3xHA) in head extracts from neuronal and glial transgenic male flies aged 21 days at 29°C depicted in Figure 2A (neuronal) and 3A (glial). Ponceau-S stained blot revealing all transferred proteins is shown below.

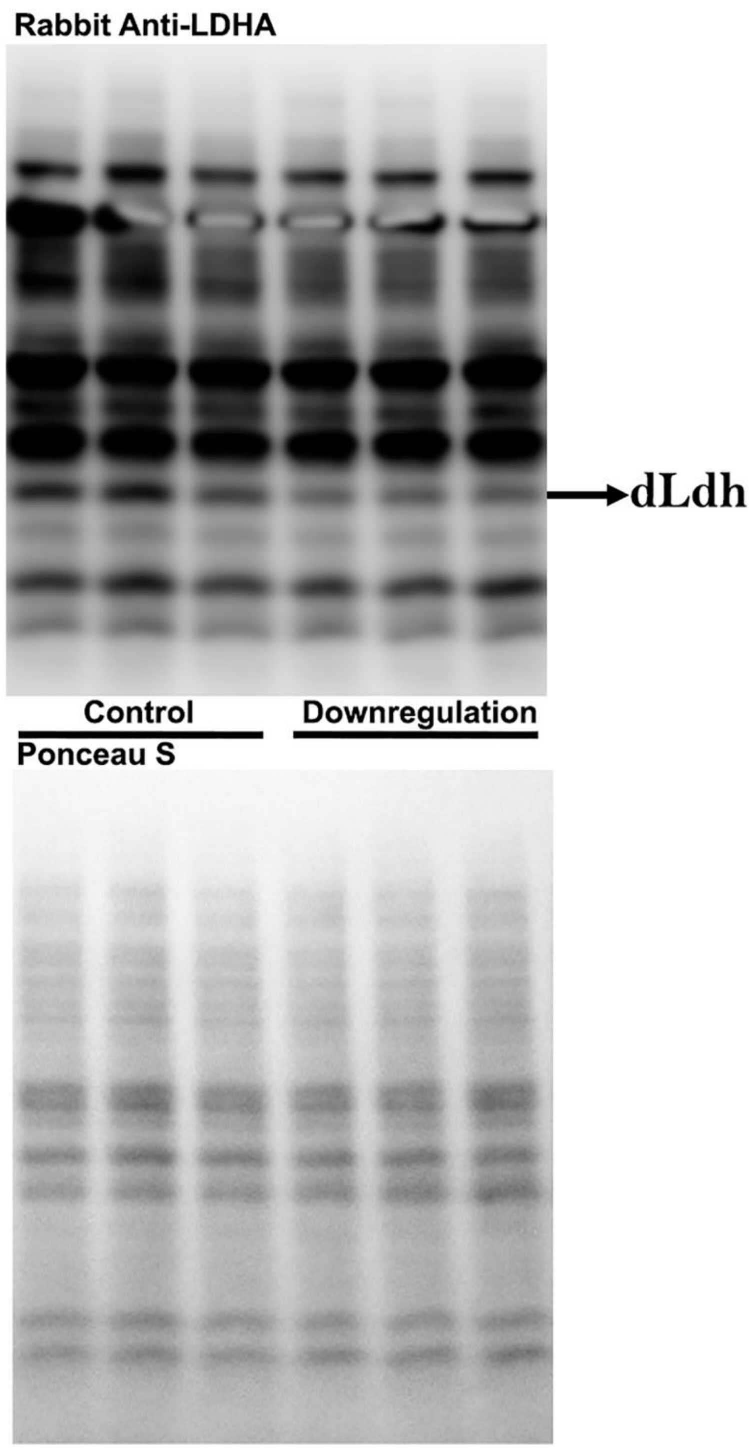

Supplementary Figure 4. Full Western blot images for Figure 3A dLdh detection. Full images for western blot analysis to detect dLdh (~35.5kD) in head extracts from glial transgenic male flies aged 21 days at 29°C quantified in Figure 3A. Ponceau-S stained blot revealing all transferred proteins is shown below.

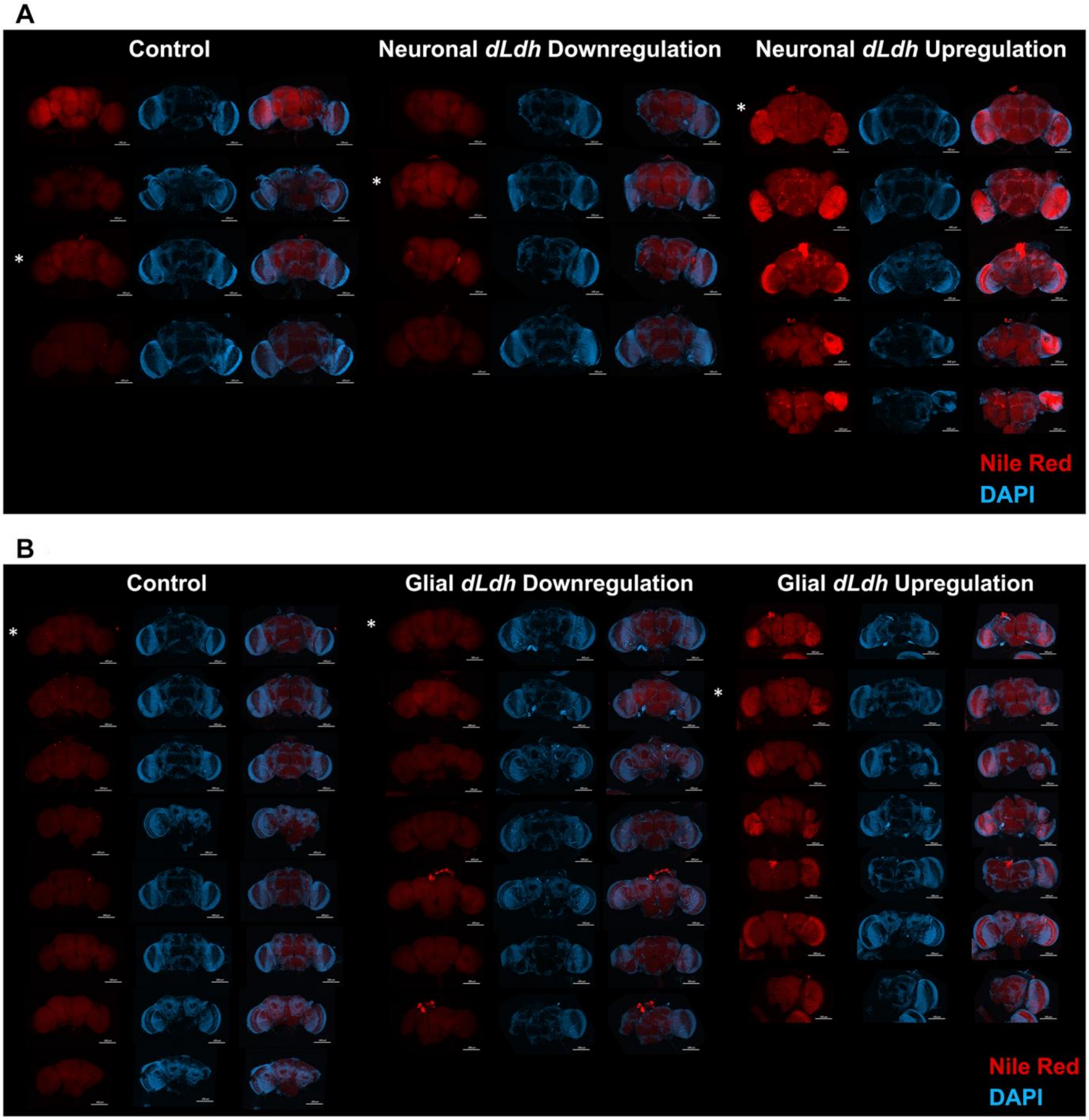

Supplementary Figure 5. Neutral lipid staining of all 21 day aged male transgenic fly whole brains with altered glial or neuronal *dLdh* expression. (**A**) All confocal fluorescence microscopy images of neuronal *dLdh* transgenic male fly brains removed at 21 days of age at 29°C and stained for DNA (Blue = DAPI) and neutral lipids (Red = Nile Red). (**B**) All confocal fluorescence microscopy images of glial *dLdh* transgenic male fly brains removed at 21 days of age at 29°C and stained for DNA (Blue = DAPI) and neutral lipids (Red = Nile Red).

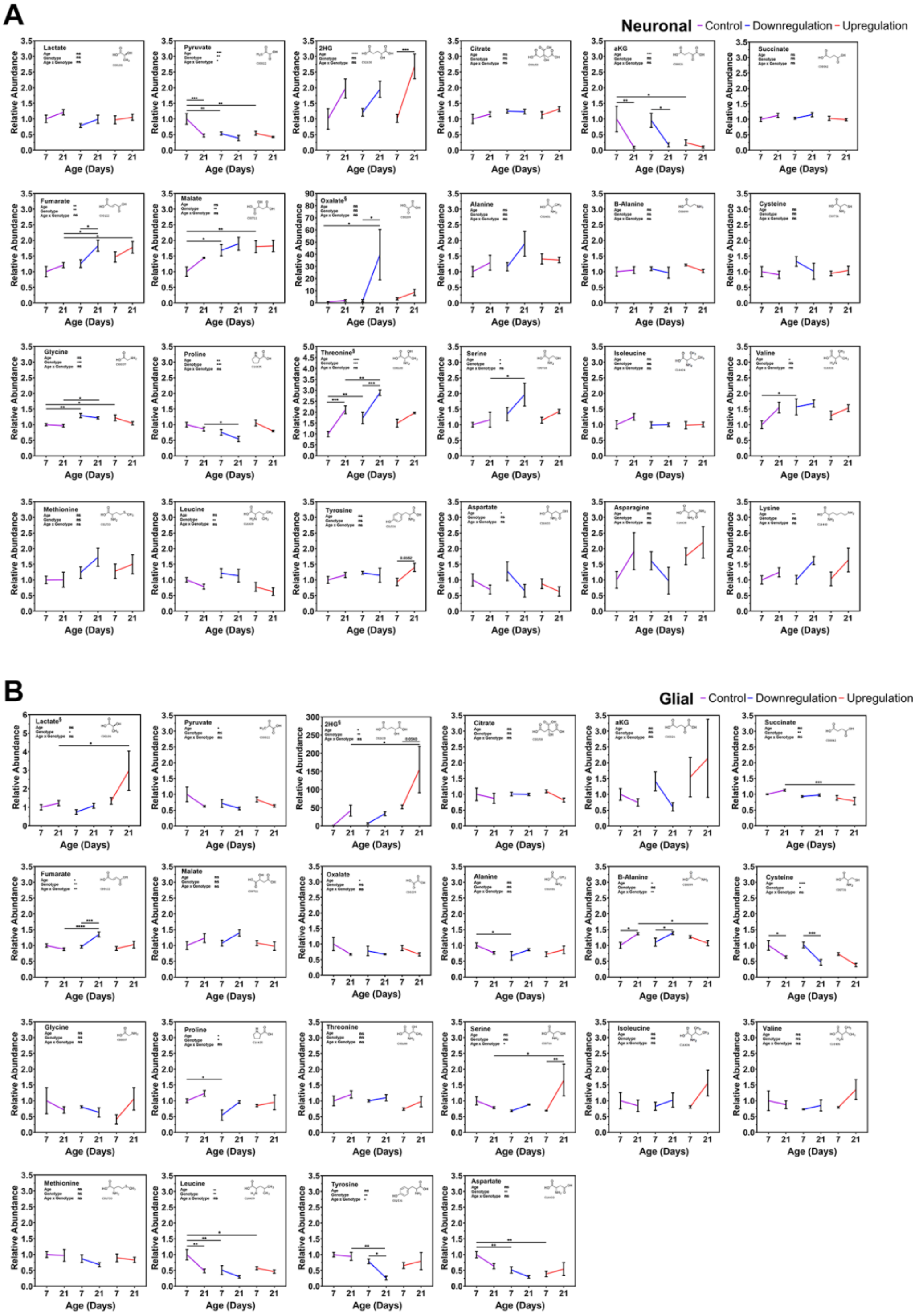

Supplementary Figure 6. Expanded panel of metabolites detected in transgenic male fly heads aged 7 and 21 days with altered expression of neuronal or glial *dLdh*. (**A**) All metabolites measured by gas chromatography-mass spectrometry (GC-MS) in the heads of neuronal *dLdh* transgenic male flies aged 7 and 21 days at 29°C (**B**) All metabolites measured by gas chromatography-mass spectrometry (GC-MS) in the heads of glial *dLdh* transgenic male flies aged 7 and 21 days at 29°C. Comparisons for each metabolite were done between genotype and age groups of neuronal and glial *dLdh* transgenic flies separately using two-way ANOVAs with Dunnet's multiple comparisons with control for each age group and with Šídák's multiple comparisons between age groups within each genotype. Effects of age, genotype, and age by genotype interaction are denoted on the top left of each graph. The structural formula for each metabolite were obtained from the Kyoto Encyclopedia of Genes and Genomes (KEGG) chemical compound database with associated C number below.
